# Consistent Estimation of Generalized Linear Models with High Dimensional Predictors via Stepwise Regression

**DOI:** 10.3390/e22090965

**Published:** 2020-08-31

**Authors:** Alex Pijyan, Qi Zheng, Hyokyoung G. Hong, Yi Li

**Affiliations:** 1 Department of Statistics and Probability, Michigan State University, East Lansing, MI 48824, USA; pijyanal@msu.edu; 2 Department of Bioinformatics and Biostatistics, University of Louisville, Louisville, KY 40202, USA; qi.zheng@louisville.edu; 3 Department of Biostatistics, University of Michigan, Ann Arbor, MI 48109, USA; yili@umich.edu

**Keywords:** estimation consistency, generalized linear models, high dimensional predictors, model selection, stepwise regression

## Abstract

Predictive models play a central role in decision making. Penalized regression approaches, such as least absolute shrinkage and selection operator (LASSO), have been widely used to construct predictive models and explain the impacts of the selected predictors, but the estimates are typically biased. Moreover, when data are ultrahigh-dimensional, penalized regression is usable only after applying variable screening methods to downsize variables. We propose a stepwise procedure for fitting generalized linear models with ultrahigh dimensional predictors. Our procedure can provide a final model; control both false negatives and false positives; and yield consistent estimates, which are useful to gauge the actual effect size of risk factors. Simulations and applications to two clinical studies verify the utility of the method.

## 1. Introduction

In the era of precision medicine, constructing interpretable and accurate predictive models, based on patients’ demographic characteristics, clinical conditions and molecular biomarkers, has been crucial for disease prevention, early diagnosis and targeted therapy [1]. When the number of predictors is moderate, penalized regression approaches such as least absolute shrinkage and selection operator (LASSO) by [2] have been used to construct predictive models and explain the impacts of the selected predictors. However, in ultrahigh dimensional settings where *p* is in the exponential order of *n*, penalized methods may incur computational challenges [3], may not reach globally optimal solutions and often generate biased estimates [4]. Sure independence screening (SIS) proposed by [5] has emerged as a powerful tool for modeling ultrahigh dimensional data. However, the method relies on a partial faithfulness assumption, which stipulates that jointly important variables must be marginally important, an assumption that may not be always realistic. To relieve this condition, some iterative procedures, such as ISIS [5], have been adopted to repeatedly screen variables based on the residuals from the previous iterations, but with heavy computation and unclear theoretical properties. Conditional screening approaches [see, e.g., [6]] have, to some extent, addressed the challenge. However, screening methods do not directly generate a final model, and post-screening regularization methods, such as LASSO, are recommended by [5] to produce a final model.

For generating a final predictive model in ultrahigh dimensional settings, recent years have seen a surging interest of performing forward regression, an old technique for model selection; see [7,8,9], among many others. Under some regularity conditions and with some proper stopping criteria, forward regression can achieve screening consistency and sequentially select variables according to metrics such as AIC, BIC or $R^{2}$. Closely related to forward selection also, is least angle regression (LARS) [10], a widely used model selection algorithm for high-dimensional models. In the generalized linear model setting [11,12], proposed differential geometrical LARS (dgLARS) based on a differential geometrical extension of LARS.

However, these methods have drawbacks. First, once a variable is identified by the forward selection, it is not removable from the list of selected variables. Hence, false positives are unavoidable without a systematic elimination procedure. Second, most of the existing works focus on variable selection and are silent with respect to estimation accuracy.

To address the first issue, some works have been proposed to add backward elimination steps once forward selection is accomplished, as backward elimination may further eliminate false positives from the variables selected by forward selection. For example, ref. [13,14] proposed a stepwise selection for linear regression models in high-dimensional settings and proved model selection consistency. However, it is unclear whether the results hold for high-dimensional generalized linear models (GLMs); Ref. [15] proposed a similar stepwise algorithm in high-dimensional GLM settings, but with no theoretical properties on model selection. Moreover, none of the relevant works have touched upon the accuracy of estimation.

We extend a stepwise regression method to accommodate GLMs with high-dimensional predictors. Our method embraces both model selection and estimation. It starts with an empty model or pre-specified predictors, scans all features and sequentially selects features, and conducts backward elimination once forward selection is completed. Our proposal controls both false negatives and false positives in high dimensional settings: the forward selection steps recruit variables in an inclusive way by allowing some false positives for the sake of avoiding false negatives, while the backward selection steps eliminate the potential false positives from the recruited variables. We use different stopping criteria in the forward and backward selection steps, to control the numbers of false positives and false negatives. Moreover, we prove that, under a sparsity assumption of the true model, the proposed approach can discover all of the relevant predictors within a finite number of steps, and the estimated coefficients are consistent, a property still unknown to the literature. Finally, our GLM framework enables our work to accommodate a wide range of data types, such as binary, categorical and count data.

To recap, our proposed method distinguishes from the existing stepwise approaches in high dimensional settings. For example, it improves  [13,14] by extending the work to a more broad GLM setting and  [15] by establishing the theoretical properties.

Compared with the other variable selection and screening works, our method produces a final model in ultrahigh dimensional settings, without applying a pre-screening step which may produce unintended false negatives. Under some regularity conditions, the method identifies or includes the true model with probability going to 1. Moreover, unlike the penalized approaches such as LASSO, the coefficients estimated by our stepwise selection procedure in the final model will be consistent, which are useful for gauging the real effect sizes of risk factors.

## 2. Method

Let $(X_{i},Y_{i}),\;i=1,\ldots ,n,$ denote *n* independently and identically distributed (i.i.d.) copies of (X,Y). Here, $X={\left(1,X_{1},\ldots ,X_{p}\right)}^{T}$ is a (p+1)-dimensional predictor vector with $X_{0}=1$ corresponding to the intercept term, and *Y* is an outcome. Suppose that the conditional density of *Y*, given X, belongs to a linear exponential family:

(1) $$\begin{array}{c} \pi \left(Y|X\right)=\exp\{YX^{T}\beta -b\left(X^{T}\beta \right)+A\left(Y\right)\}, \end{array}$$

where $\beta ={\left(\beta_{0},\beta_{1},\ldots ,\beta_{p}\right)}^{T}$ is the vector of coefficients; $\beta_{0}$ is the intercept; and A(·) and b(·) are known functions. Model (1), with a canonical link function and a unit dispersion parameter, belongs to a larger exponential family [16]. Further, b(·) is assumed twice continuously differentiable with a non-negative second derivative $b^{\prime \prime }\left(\;\cdot \;\right)$. We use μ(·) and σ(·) to denote $b^{\prime }\left(\;\cdot \;\right)$ and $b^{\prime \prime }\left(\;\cdot \;\right)$, i.e., the mean and variance functions, respectively. For example, b(θ)=log(1+exp(θ)) in a logistic distribution and b(θ)=exp(θ) in a Poisson distribution.

Let L(u,v)=uv−b(u) and $E_{n}\left\{f\left(\xi \right)\right\}=n^{-1}\sum_{i=1}^{n}f\left(\xi_{i}\right)$ denote the mean of ${\left\{f\left(\xi_{i}\right)\right\}}_{i=1}^{n}$ for a sequence of i.i.d. random variables $\xi_{i}$(i=1,…,n) and a non-random function f(·). Based on the i.i.d. observations, the log-likelihood function is

(2) $$\begin{array}{c} \ell \left(\beta \right)=n^{-1}\sum_{i=1}^{n}L\left(X_{i}^{T}\beta ,Y_{i}\right)=E_{n}\left\{L\left(X^{T}\beta ,Y\right)\right\}. \end{array}$$

We use $\beta_{*}={\left(\beta_{*0},\beta_{*1},\ldots ,\beta_{*p}\right)}^{T}$ to denote the true values of β. Then the true model is $M=\left\{j:\beta_{*j}\neq 0,j\geq 1\right\}\cup \left\{0\right\}$, which consists of the intercept and all variables with nonzero effects. Overarching goals of ultra-high dimensional data analysis are to identify M and estimate $\beta_{*j}$ for j∈M. While most of the relevant literature [8,9] is on estimating M, this work is to accomplish both identification of M and estimation of $\beta_{*j}$.

When *p* is in the exponential order of *n*, we aim to generate a predictive model that contains the true model with high probability, and provide consistent estimates of regression coefficients. We further introduce the following notation. For a generic index set S⊂{0,1,…,p} and a (p+1)-dimensional vector A, we use $S^{c}$ to denote the complement of a set *S* and $A_{S}=\left\{A_{j}:j\in S\right\}$ to denote the subvector of A corresponding to *S*. For instance, if S={2,3,4}, then $X_{iS}={\left(X_{i2},X_{i3},X_{i4}\right)}^{T}$. Moreover, denote by $\ell_{S}\left(\beta_{S}\right)=E_{n}\left\{L\left(X_{S}^{T}\beta_{S},Y\right)\right\}$ the log-likelihood of the regression model of *Y* on $X_{S}$ and denote by ${\hat{\beta }}_{S}$ the maximizer of $\ell_{S}\left(\beta_{S}\right)$. Under model (1), we elaborate on the idea of stepwise (details in the supplementary materials) selection, consisting of the forward and backward stages.

**Forward stage:** We start with $F_{0}$, a set of variables that need to be included according to some *a priori* knowledge, such as clinically important factors and conditions. If no such information is available, $F_{0}$ is set to be {0}, corresponding to a null model. We sequentially add covariates as follows:

$$F_{0}\subset F_{1}\subset F_{2}\subset \cdots \subset F_{k},$$

where $F_{k}\subset \left\{0,1,\ldots ,p\right\}$ is the index set of the selected covariates upon completion of the *k*th step, with k≥0. At the (k+1)th step, we append new variables to $F_{k}$ one at a time and refit GLMs: for every $j\in F_{k}^{c}$, we let $F_{k,j}=F_{k}\cup \left\{j\right\}$, obtain ${\hat{\beta }}_{F_{k,j}}$ by maximizing $\ell_{F_{k,j}}\left(\beta_{F_{k,j}}\right)$, and compute the increment of log-likelihood,

$$\ell_{F_{k,j}}\left({\hat{\beta }}_{F_{k,j}}\right)-\ell_{F_{k}}\left({\hat{\beta }}_{F_{k}}\right).$$

Then the index of a new candidate variable is determined to be

$$j_{k+1}=\underset{j\in F_{k}^{c}}{arg max}\ell_{F_{k,j}}\left({\hat{\beta }}_{F_{k,j}}\right)-\ell_{F_{k}}\left({\hat{\beta }}_{F_{k}}\right).$$

Additionally, we update $F_{k+1}=F_{k}\cup \left\{j_{k+1}\right\}.$ We then need to decide whether to stop at the *k*th step or move on to the (k+1)th step with $F_{k+1}$. To do so, we use the following EBIC criterion:

(3) $$EBIC\left(F_{k+1}\right)=-2\ell_{F_{k+1}}\left({\hat{\beta }}_{F_{k+1}}\right)+\left|F_{k+1}\right|n^{-1}\left(\log n+2\eta_{1}\log p\right),$$

where the second term is motivated by [17] and |F| denotes the cardinality of a set *F*.

The forward selection stops if $EBIC\left(F_{k+1}\right)>EBIC\left(F_{k}\right)$. We denote the stopping step by $k^{*}$ and the set of variables selected so far by $F_{k^{*}}$.

**Backward stage:** Upon the completion of forward stage, backward elimination, starting with $B_{0}=F_{k^{*}}$, sequentially drops covariates as follows:

$$B_{0}⊃B_{1}⊃B_{2}⊃\cdots ⊃B_{k},$$

where $B_{k}$ is the index set of the remaining covariates upon the completion of the *k*th step of the backward stage, with k≥0. At the (k+1)th backward step and for every $j\in B_{k}$, we let $B_{k/j}=B_{k}\backslash \left\{j\right\}$, obtain ${\hat{\beta }}_{B_{k/j}}$ by maximizing $\ell (\beta_{B_{k/j}})$, and calculating the difference of the log-likelihoods between these two nested models:

$$\ell_{B_{k}}\left({\hat{\beta }}_{B_{k}}\right)-\ell_{B_{k/j}}\left({\hat{\beta }}_{B_{k/j}}\right).$$

The variable that can be removed from the current set of variables is indexed by

$$j_{k+1}=\underset{j\in B_{k}}{arg min}\ell_{B_{k}}\left({\hat{\beta }}_{B_{k}}\right)-\ell_{B_{k/j}}\left({\hat{\beta }}_{B_{k/j}}\right).$$

Let $B_{k+1}=B_{k}\backslash \left\{j_{k+1}\right\}.$ We determine whether to stop at the *k*th step or move on to the (k+1)th step of the backward stage according to the following BIC criterion:

(4) $$BIC\left(B_{k+1}\right)=-2\ell_{B_{k+1}}\left({\hat{\beta }}_{B_{k+1}}\right)+\eta_{2}n^{-1}\left|B_{k+1}\right|\log n.$$

If $BIC\left(B_{k+1}\right)>BIC\left(B_{k}\right)$, we end the backward stage at the *k*th step. Let $k^{**}$ denote the stopping step and we declare the selected model $B_{k^{**}}$ to be the final model. Thus, $\hat{M}=B_{k^{**}}$ is the estimate of M. As the backward stage starts with the $k^{*}$ variables selected by forward selection, $k^{**}$ cannot exceed $k^{*}$.

A strength of our algorithm, termed STEPWISE hereafter, is the added flexibility with $\eta_{1}$ and $\eta_{2}$ in the stopping criteria for controlling the false negatives and positives. For example, a smaller value of $\eta_{1}$ close to zero in the forward selection step will likely include more variables, and thus incur more false positives and less false negatives, whereas a larger value of $\eta_{1}$ will recruit too few variables and cause too many false negatives. Similarly, in the backward selection step, a large $\eta_{2}$ would eliminate more variables and therefore further reduce more false positives, and vice versa for a small $\eta_{2}$. While finding optimal $\eta_{1}$ and $\eta_{2}$ is not trivial, our numerical experiences suggest a small $\eta_{1}$ and a large $\eta_{2}$ may well balance the false negatives and positives. When $\eta_{2}=0$, no variables can be dropped after forward selection; hence, our proposal includes forward selection as a special case.

Moreover, [8] proposed a sequentially conditioning approach based on offset terms that absorb the prior information. However, our numerical experiments indicate that the offset approach may be suboptimal compared to our full stepwise optimization approach, which will be demonstrated in the simulation studies.

## 3. Theoretical Properties

With a column vector v, let ${∥v∥}_{q}$ denote the $L_{q}$-norm for any q≥1. For simplicity, we denote the $L_{2}$-norm of v by ∥v∥, and denote $vv^{T}$ by $v^{⊗2}$. We use $C_{1},C_{2},\ldots ,$ to denote some generic constants that do not depend on *n* and may change from line to line. The following regularity conditions are set.

1. There exist a positive integer *q* satisfying |M|≤q and $q\log p=o(n^{1/3})$ and a constant K>0 such that $\sup_{|S|\leq q}{∥\beta_{S}^{*}∥}_{1}\leq K$, where $\beta_{S}^{*}={arg max}_{\beta_{S}}E\left[\ell_{S}\left(\beta_{S}\right)\right]$ is termed the least false value of model *S*.
2. ${∥X∥}_{\infty }\leq K$. In addition, $E(X_{j})=0$ and $E(X_{j}^{2})=1$ for j≥1.
3. Let $\epsilon_{i}=Y_{i}-\mu \left(\beta_{*}^{T}X_{i}\right)$. There exists a positive constant *M* such that the Cramer condition holds, i.e., $E\left[{\left|\epsilon_{i}\right|}^{m}\right]\leq m!M^{m}$ for all m≥1.
4. |σ(a)−σ(b)|≤K|a−b| and $\sigma_{\min}:=\inf_{\left|t\right|\leq K^{3}}\left|b^{\prime \prime }\left(t\right)\right|$ is bounded below.
5. There exist two positive constants, $\kappa_{\min}$ and $\kappa_{\max}$ such that $0<\kappa_{\min}<\Lambda \left(E\left(X_{S}^{⊗2}\right)\right)<\kappa_{\max}<\infty$, uniformly in S⊂{0,1,…,p} satisfying |S|≤q, where Λ(A) is the collection of all eigenvalues of a square matrix A.
6. $\min_{S:M⊈S,|S|\leq q}D_{S}>Cn^{-\alpha }$ for some constants C>0 and α>0 that satisfies $qn^{-1+4\alpha }\log p\to 0$, where $D_{S}=\max_{j\in S^{c}\cap M}\left|E\left[\left(\mu \left(\beta_{*}^{T}X\right)-\mu \left(\beta_{S}^{*T}X_{S}\right)\right)X_{j}\right]\right|$.

Condition (1), as assumed in  [8,18], is an alternative to the Lipschitz assumption [5,19]. The bound of the model size allowed in the selection procedure or *q* is often required in model-based screening methods see, e.g., [8,20,21,22]. The bound should be large enough so that the correct model can be included, but not too large; otherwise, excessive noise variables would be included, leading to unstable and inconsistent estimates. Indeed, Conditions (1) and (6) reveal that the range of *q* depends on the true model size |M|, the minimum signal strength, $n^{-\alpha }$ and the total number of covariates, *p*. The upper bound of *q* is $o(\left(n^{1-4\alpha }/\log p\right)\wedge \left(n^{1/3}/\log p\right))$, ensuring the consistency of EBIC [17]. Condition (1) also implies that the parameter space under consideration can be restricted to $B:=\{\beta \in R^{p+1}:∥\beta {∥}_{1}\leq K^{2}\}$, for any model *S* with |S|≤q. Condition (2), as assumed in  [23,24], reflects that data are often standardized at the pre-processing stage. Condition (3) ensures that *Y* has a light tail, and is satisfied by Gaussian and discrete data, such as binary and count data [25]. Condition (4) is satisfied by common GLM models, such as Gaussian, binomial, Poisson and gamma distributions. Condition (5) represents the sparse Riesz condition [26] and Condition (6) is a strong "irrepresentable" condition, suggesting that M cannot be represented by a set of variables that does not include the true model. It further implies that adding a signal variable to a mis-specified model will increase the log-likelihood by a certain lower bound [8]. The signal rate is comparable to the conditions required by the other sequential methods, see, e.g., [7,22].

Theorem 1 develops a lower bound of the increment of the log-likelihood if the true model M is not yet included in a selected model *S*.

**Theorem** **1.** *Suppose Conditions (1)–(6) hold. There exists some constant $C_{1}$ such that with probability at least 1–6exp(−6qlogp),*

$$\begin{array}{c} \min_{S:M⊈S,|S|<q}\left\{\max_{j\in S^{c}}\ell_{S\cup \{j\}}\left({\hat{\beta }}_{S\cup \{j\}}\right)-\ell_{S}\left({\hat{\beta }}_{S}\right)\right\}\geq C_{1}n^{-2\alpha }. \end{array}$$

Theorem 1 shows that, before the true model is included in the selected model, we can append a variable which will increase the log-likelihood by at least $C_{1}n^{-2\alpha }$ with probability tending to 1. This ensures that in the forward stage, our proposed STEPWISE approach will keep searching for signal variables until the true model is contained. To see this, suppose at the *k*th step of the forward stage that $F_{k}$ satisfies $M⊈F_{k}$ and $|F_{k}|<q$, and let *r* be the index selected by STEPWISE. By Theorem 1, we obtain that, for any $\eta_{1}>0$, when *n* is sufficiently large,

$$\begin{array}{cc} \mathrm{EBIC}\left(F_{k,r}\right)-\mathrm{EBIC}\left(F_{k}\right)\; & =-2\ell_{F_{k,r}}\left({\hat{\beta }}_{F_{k,r}}\right)+\left(\right|F_{k}\left|+1\right)n^{-1}\left(\log n+2\eta_{1}\log p\right)\\ \; & \;\;-\left[-2\ell_{F_{k}}\left({\hat{\beta }}_{F_{k}}\right)+\left|F_{k}\right|n^{-1}\left(\log n+2\eta_{1}\log p\right)\right]\\ \; & \leq -2C_{1}n^{-2\alpha }+n^{-1}\left(\log n+2\eta_{1}\log p\right)<0, \end{array}$$

with probability at least 1−6exp(−6qlogp), where the last inequality is due to Condition (6). Therefore, with high probability the forward stage of STEPWISE continues as long as $M⊈F_{k}\;\mathrm{and}\;\left|F_{k}\right|<q$. We next establish an upper bound of the number of steps in the forward stage needed to include the true model.

**Theorem** **2.** *Under the same conditions as in Theorem 1 and if*

$$\max_{S:|S|\leq q}\left\{\max_{j\in S^{c}\cap M^{c}}\left|E\left[\left\{Y-\mu (\beta_{S}^{*T}X_{S})\right\}X_{j}\right]\right|\right\}=o\left(n^{-\alpha }\right),$$

*then there exists some constant $C_{2}>2$ such that $M\subset F_{k}$, for some $F_{k}$ in the forward stage of STEPWISE and $k\leq C_{2}\left|M\right|$, with probability at least 1−18exp(−4qlogp).*

The "max" condition, as assumed in Section 5.3 of  [27], relaxes the partial orthogonality assumption that $X_{M^{c}}$ are independent of $X_{M}$, and ensures that with probability tending to 1, appending a signal variable increases log-likelihood more than adding a noise variable does, uniformly over all possible models *S* satisfying M⊈S,|S|<q. This entails that the proposed procedure is much more likely to select a signal variable, in lieu of a noise variable, at each step. Since EBIC is a consistent model selection criterion [28,29], the following theorem guarantees termination of the proposed procedure with $M\subset F_{k}$ for some *k*.

**Theorem** **3.** *Under the same conditions as in Theorem 2 and if $M⊄F_{k-1}$ and $M\subset F_{k}$, the forward stage stops at the kth step with probability going to 1−exp(−3qlogp).*

Theorem 3 ensures that the forward stage of STEPWISE will stop within a finite number of steps and will cover the true model with probability at least 1−qexp(−3qlogp)≥1−exp(−2qlogp). We next consider the backward stage and provide a probability bound of removing a signal from a set in which the set of true signals M is contained.

**Theorem** **4.** *Under the same conditions as in Theorem 2, BIC(S\{r})−BIC(S)>0 uniformly over r∈M and S satisfying M⊂S and |S|≤q, with probability at least 1−6exp(−6qlogp).*

Theorem 4 indicates that with probability at 1−6exp(−6qlogp), BIC would decrease when removing a signal variable from a model that contains the true model. That is, with high probability, back elimination is to reduce false positives.

Recall that $F_{k^{*}}$ denotes the model selected at the end of the forward selection stage. By Theorem 2, $M\subset F_{k^{*}}$ with probability at least 1−18exp(−4qlogp). Then Theorem 4 implies that at each step of the backward stage, a signal variable will not be removed from the model with probability at least 1−6exp(−6qlogp). By Theorem 2, $|F_{k^{*}}|\leq C_{2}\left|M\right|$. Thus, the backward elimination will carry out at most $\left(C_{2}-1)|M\right|$ steps. Combining results from Theorems 2 and 3 yields that $M\subset \hat{M}$ with probability at least $1-18\exp\left(-4q\log p\right)-6\left(C_{2}-1\right)\left|M\right|\exp\left(-6q\log p\right)$. Let $\hat{\beta }$ be the estimate of $\beta_{*}$ in model (1) at the termination of STEPWISE. By convention, the estimates of the coefficients of the unselected covariates are 0.

**Theorem** **5.** *Under the same conditions as in Theorem 2, we have that $M\subseteq \hat{M}$ and*

$$∥\hat{\beta }-\beta_{*}∥\to 0$$

*in probability.*

The theorem warrants that the proposed STEPWISE yields consistent estimates, a property not shared by many regularized methods, including LASSO. Our later simulations verified this. Proof of main theorems and lemmas are provided in Appendix A.

## 4. Simulation Studies

We compared the proposal with the other competing methods, including the penalized methods, such as least absolute shrinkage and selection operator (LASSO); the differential geometric least angle regression (dgLARS) [11,12]; the forward regression (FR) approach [7]; the sequentially conditioning (SC) approach [8]; and the screening methods, such as sure independence screening (SIS) [5], which is popular in practice. As SIS does not directly generate a predictive model, we applied LASSO for the top [n/log(n)] variables chosen by SIS and denoted the procedure by SIS+LASSO. As the FR, SC and STEPWISE approaches involve forward searching and to make them comparable, we applied the same stopping rule, for example, Equation (3) with the same γ, to their forward steps. In particular, the STEPWISE approach, with $\eta_{1}=\gamma$ and $\eta_{2}=0$, is equivalent to FR and asymptotically equivalent to SC. By varying γ in FR and SC between $\gamma_{L}$ and $\gamma_{H}$, we explored the impact of γ on inducing false positives and negatives. In our numerical studies, we fixed $\gamma_{H}=10$ and set $\gamma_{L}=\eta_{1}$. To choose $\eta_{1}$ and $\eta_{2}$ in (3) and (4) in STEPWISE, we performed 5-fold cross-validation to minimize the mean squared prediction error (MSPE), and reported the results in Table 1. Since the proposed STEPWISE algorithm uses the (E)BIC criterion, for a fair comparison we chose the tuning parameter in dgLARS by using the BIC criterion as well, and coined the corresponding approach as dgLARS(BIC). The regularization parameter in LASSO was chosen via the following two approaches: (1) giving the smallest BIC for the models on the LASSO path, denoted by LASSO(BIC); (2) using the one-standard-error rule, denoted by LASSO(1SE), which chooses the most parsimonious model whose error is no more than one standard error above the error of the best model in cross-validation [30].

Denote by $X_{i}={\left(X_{i1},\ldots ,X_{ip}\right)}^{T}$ and $\beta ={\left(\beta_{1},\ldots ,\beta_{p}\right)}^{T}$, the covariate vector for subject i(1,…,n) and the true coefficient vector. The following five examples generated $X_{i}^{T}\beta$, the inner product of the coefficient and covariate vectors for each individual, which were used to generate outcomes from the normal, binomial and Poisson models.

**Example** **1.** *For each i,*

$$cX_{i}^{T}\beta =c\times \left(\sum_{j=1}^{p_{0}}\beta_{j}X_{ij}+\sum_{j=p_{0}+1}^{p}\beta_{j}X_{ij}\right),\;i=1,\ldots ,n,$$

*where $\beta_{j}$ = ${\left(-1\right)}^{B_{j}}$(4logn/$\sqrt{n}$ + $|Z_{j}|$), for $j=1,\ldots ,p_{0}$ and $\beta_{j}=$0 otherwise $B_{j}$ was a binary random variable with $P(B_{j}=1)=0.4$ and $Z_{j}$ was generated by a standard normal distribution; $p_{0}=8$; $X_{ij}$s were independently generated from a standardized exponential distribution, that is, exp(1)−1. Here and also in the other examples, c (specified later) controls the signal strengths.*

**Example** **2.** *This scenario is the same as* **Example 1** *except that $X_{ij}$ was independently generated from a standard normal distribution.*

**Example** **3.** *For each i,*

$$cX_{i}^{T}\beta =c\times \left(\sum_{j=1}^{p_{0}}\beta_{j}X_{ij}+\sum_{j=p_{0}+1}^{p}\beta_{j}X_{ij}^{*}\right),\;i=1,\ldots ,n,$$

where $\beta_{j}$ = 2j for 1 ≤j≤$p_{0}$ and $p_{0}=5$. We simulated every component of $Z_{i}$ = ($Z_{ij}$) ∈$R^{p}$ and $W_{i}$ = ($W_{ij}$) ∈$R^{p}$ independently from a standard normal distribution. Next, we generated $X_{i}$ according to $X_{ij}$ = $\left(Z_{ij}+W_{ij}\right)/\sqrt{2}$ for 1 ≤j≤$p_{0}$ and $X_{ij}^{*}=\left(Z_{ij}+\sum_{j^{\prime }=1}^{p_{0}}Z_{ij^{\prime }}\right)/2$ for $p_{0}<j\leq p$.

**Example** **4.** *For each i,*

$$cX_{i}^{T}\beta =c\times \left(\sum_{j=1}^{500}\beta_{j}X_{ij}+\sum_{j=501}^{p}\beta_{j}X_{ij}\right),\;i=1,\ldots ,n,$$

where the first 500 $X_{ij}$s were generated from the multivariate normal distribution with mean 0 and a covariance matrix with all of the diagonal entries being 1 and $cov(X_{ij},X_{ij^{\prime }})$ = $0.5^{|j-j^{\prime }|}$ for $1\leq j,j^{\prime }\leq p$. The remaining p−500$X_{ij}$s were generated through the autoregressive processes with $X_{i,501}$∼ Unif(-2, 2), $X_{ij}$ = 0.5$X_{i,j-1}$ + 0.5$X_{ij}^{*}$, for j=502,…,p, where $X_{ij}^{*}$∼ Unif(-2, 2) were generated independently. The coefficients $\beta_{j}$ for j=1,…,7,501,…,507 were generated from ${\left(-1\right)}^{B_{j}}$(4logn/$\sqrt{n}$ + $|Z_{j}|$), where $B_{j}$ was a binary random variable with $P(B_{j}=1)=0.4$ and $Z_{j}$ was from a standard normal distribution. The remaining $\beta_{j}$ were zeros.

**Example** **5.** *For each i,*

$$cX_{i}^{T}\beta =c\times \left(-0.5X_{i1}+X_{i2}+0.5X_{i,100}\right),\;i=1,\ldots ,n,$$

*where $X_{i}$ were generated from a multivariate normal distribution with mean 0 and a covariance matrix with all of the diagonal entries being 1 and $cov(X_{ij},X_{ij^{\prime }})$ = $0.9^{|j-j^{\prime }|}$ for 1 ≤ j, j’ ≤ p. All of the coefficients were zero except for $X_{i1}$, $X_{i2}$ and $X_{i,100}$.*

**Examples 1 and 3** were adopted from [7], while **Examples 2 and 4** were borrowed from [5,15], respectively. We then generated the responses from the following three models.

**Normal model:**$Y_{i}=cX_{i}^{T}\beta +\epsilon_{i}$ with $\epsilon_{i}∼N\left(0,1\right)$.

**Binomial model:**$Y_{i}∼$ Bernoulli( $\exp\left(cX_{i}^{T}\beta \right)/\left\{1+\exp\left(cX_{i}^{T}\beta \right)\right\})$.

**Poisson model:**$Y_{i}∼$ Poisson( $\exp(cX_{i}^{T}\beta )$).

We considered *n* = 400 and *p* = 1000 throughout all of the examples. We specified the magnitude of the coefficients in the GLMs with a constant multiplier, *c*. For Examples 1–5, this constant was set, respectively for the normal, binomial and Poisson models, to be: (1, 1, 0.3), (1, 1.5, 0.3), (1, 1, 0.1), (1, 1.5, 0.3) and (1, 3, 2). For each parameter configuration, we simulated 500 independent data sets. We evaluated the performances of the methods by the criteria of true positives (TP), false positives (FP), the estimated probability of including the true models (PIT), the mean squared error (MSE) of $\hat{\beta }$ and the mean squared prediction error (MSPE). To compute the MSPE, we randomly partitioned the samples into the training (75%) and testing (25%) sets. The models obtained from the training datasets were used to predict the responses in the testing datasets. Table 2, Table 3 and Table 4 report the average TP, FP, PIT, MSE and MSPE over 500 datasets along with the standard deviations. The findings are summarized below.

First, the proposed STEPWISE method was able to detect all the true signals with nearly zero FPs. Specifically, in all of the Examples, STEPWISE outperformed the other methods by detecting more TPs with fewer FPs, whereas LASSO, SIS+LASSO and dgLARS included much more FPs.

Second, though a smaller γ in FR and SC led to the inclusion of all TPs with a PIT close to 1, it incurred more FPs. On the other hand, a larger γ may eliminate some TPs, resulting in a smaller PIT and a larger MSPE.

Third, for the normal model, the STEPWISE method yielded an MSE close to 0, the smallest among all the competing methods. The binary and Poisson data challenged all of the methods, and the MSEs for all the methods were non-negligible. However, the STEPWISE method still produced the lowest MSE. The results seemed to verify the consistency of $\hat{\beta }$, which distinguished the proposed STEPWISE method from the other regularized methods and highlighted its ability to provide a more accurate means to characterize the effects of high dimensional predictors.

## 5. Real Data Analysis

### 5.1. A Study of Gene Regulation in the Mammalian Eye

To demonstrate the utility of our proposed method, we analyzed a microarray dataset from [35] with 120 twelve-week male rats selected for eye tissue harvesting. The dataset contained more than 31,042 different probe sets (Affymetric GeneChip Rat Genome 230 2.0 Array); see [35] for a more detailed description of the data.

Although our method was applicable to the original 31,042 probe sets, many probes turned out to have very small variances and were unlikely to be informative for correlative analyses. Therefore, using variance as the screening criterion, we selected 5000 genes with the largest variances in expressions and correlated them with gene *TRIM32* that has been found to cause Bardet–Biedl syndrome, a genetically heterogeneous disease of multiple organ systems including the retina [36].

We applied the proposed STEPWISE method to the dataset with n=120 and p=5000, and treated the *TRIM32* gene expression as the response variable and the expressions of 5000 genes as the predictors. With no prior biological information available, we started with the empty set. To choose $\eta_{1}$ and $\eta_{2}$, we carried out 5-fold cross-validation to minimize the mean squared prediction error (MSPE) by using the following grid search: $\eta_{1}=\left\{0,0.25,0.5,1\right\}$ and $\eta_{2}=\left\{1,2,3,4,5\right\}$, and set $\eta_{1}=1$ and $\eta_{2}=4$. We also performed the same procedure to choose the γ for FR and SC. The regularization parameters in LASSO and dgLARS were selected to minimize BIC values.

In the forward step, STEPWISE selected the probes of *1376747_at*, *1381902_at*, *1382673_at* and *1375577_at*, and the backward step eliminated probe *1375577_at*. The STEPWISE procedure produced the following final predictive model:

*TRIM32* = 4.6208 + 0.2310 × (*1376747_at*) + 0.1914 × (*1381902_at*) + 0.1263 × (*1382673_at*). Table A1 in Appendix B presents the numbers of overlapping genes among competing methods. It shows that the two out of three probes, *1381902_at* and *1376747_at*, selected from our method are also discovered by the other methods, except for dgLARS.

Next, we performed Leave-One-Out Cross-Validation (LOOCV) to obtain the distribution of the model size (MS) and MSPE for the competing methods.

**Figure 1:**
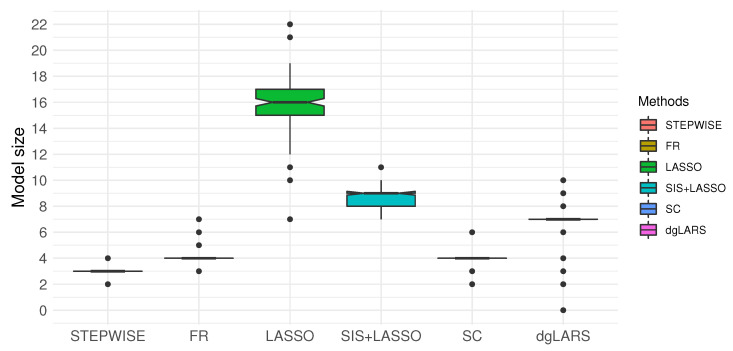
Box plot of model sizes for each method over 120 different training samples from the mammalian eye data set. STEPWISE was performed with $\eta_{1}=1$ and $\eta_{2}=4$, and FR and SC were conducted with γ=1.

As reported in Table 5 and Figure 1, LASSO, SIS+LASSO and dgLARS tended to select more variables than the forward approaches and STEPWISE. Among all of the methods, STEPWISE selected the fewest variables but with almost the same MSPE as the other methods.

### 5.2. An Esophageal Squamous Cell Carcinoma Study

Esophageal squamous cell carcinoma (ESCC), the most common histological type of esophageal cancer, is known to be associated with poor overall survival, making early diagnosis crucial for treatment and disease management [37]. Several studies have investigated the roles of circulating microRNAs (miRNAs) in diagnosis of ESCC [38].

Using a clinical study that investigated the roles of miRNAs on the ESCC [39], we aimed to use miRNAs to predict ESCC risks and estimate their impacts on the development of ESCC. Specifically, with a dataset of serum profiling of 2565 miRNAs from 566 ESCC patients and 4965 controls without cancer, we demonstrated the utility of the proposed STEPWISE method in predicting ESCC with miRNAs.

To proceed, we used a balance sampling scheme (283 cases and 283 controls) in the training dataset. The design of yielding an equal number of cases and controls in the training set has proved to be useful [39] for handling imbalanced outcomes as we encountered here. To validate our findings, samples were randomly divided into a training ($n_{1}=566$, p=2565) and testing set ($n_{2}=4965$, p=2565).

The training set consisted of 283 patients with ESCC (median age of 65 years, 79% male) and 283 control patients (median age of 68 years, 46.3% male), and the testing set consisted of 283 patients with ESCC (median age of 67 years, 85.7% male) and 4682 control patients (median age of 67.5 years, 44.5% male). Control patients without ESCC came from three sources: 323 individuals from National Cancer Center Biobank (NCCB); 2670 individuals from the Biobank of the National Center for Geriatrics and Gerontology (NCGG); and 1972 individuals from Minoru Clinic (MC). More detailed characteristics of cases and controls in the training and testing sets are given in Table 6.

We defined the binary outcome variable to be 1 if the subject was a case and 0 otherwise. As age and gender (0 = female, 1 = male) are important risk factors for ESCC [40,41] and it is common to adjust for them in clinical models, we set the initial set in STEPWISE to be $F_{0}=$ {age, gender}. With $\eta_{1}=0$ and $\eta_{2}=3.5$ that were also chosen from 5-fold CV, our procedure recruited three miRNAs. More specifically, *miR-4783-3p*, *miR-320b*, *miR-1225-3p* and *miR-6789-5p* were selected among 2565 miRNAs by the forward stage from the training set, and then the backward stage eliminated *miR-6789-5p*.

In comparison, with γ=0, both FR and SC selected four miRNAs, *miR-4783-3p*, *miR-320b*, *miR-1225-3p* and *miR-6789-5p*. The list of selected miRNAs by different methods are given in Table A2 in Appendix B.

Our findings were biologically meaningful, as the selected miRNAs had been identified by other cancer studies as well. Specifically, *miR-320b* was found to promote colorectal cancer proliferation and invasion by competing with its homologous *miR-320a* [42]. In addition, serum levels of *miR-320* family members were associated with clinical parameters and diagnosis in prostate cancer patients [43]. Reference [44] showed that *miR-4783-3p* was one of the miRNAs that could increase the risk of colorectal cancer death among rectal cancer cases. Finally, *miR-1225-5p* inhibited proliferation and metastasis of gastric carcinoma through repressing insulin receptor substrate-1 and activation of β-catenin signaling [45].

Aiming to identify a final model without resorting to a pre-screening procedure that may miss out on important biomarkers, we applied STEPWISE to reach the following predictive model for ESCC based on patients’ demographics and miRNAs:

${\mathrm{logit}}^{-1}\left(-35.70+1.41\times \mathrm{miR}-4783-3p+0.98\times \mathrm{miR}-320b+1.91\times \mathrm{miR}-1225-3p+0.10\times \mathrm{Age}-2.02\times \mathrm{Gender}\right)$, where ${\mathrm{logit}}^{-1}\left(x\right)=\exp\left(x\right)/\left(1+\exp\left(x\right)\right)$.

**Figure 2:**
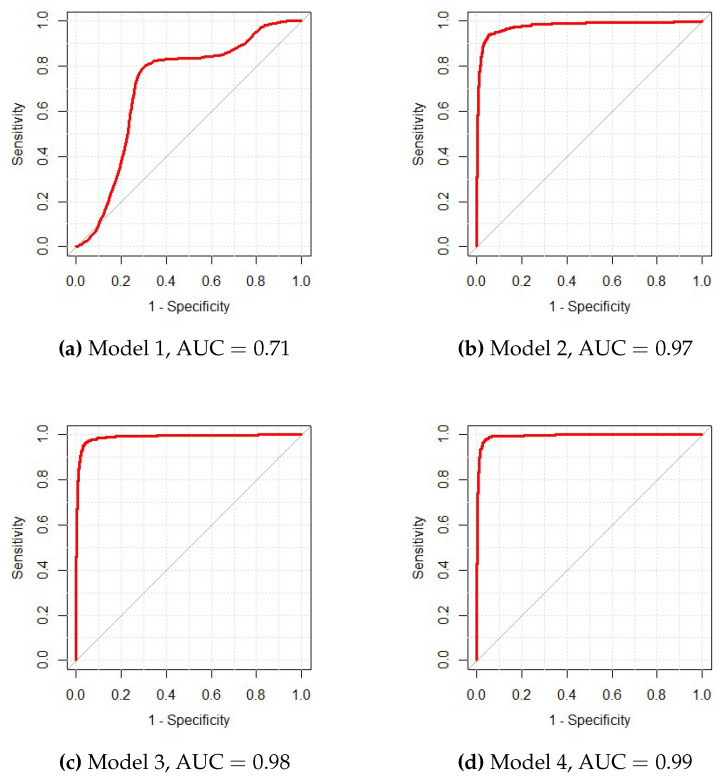

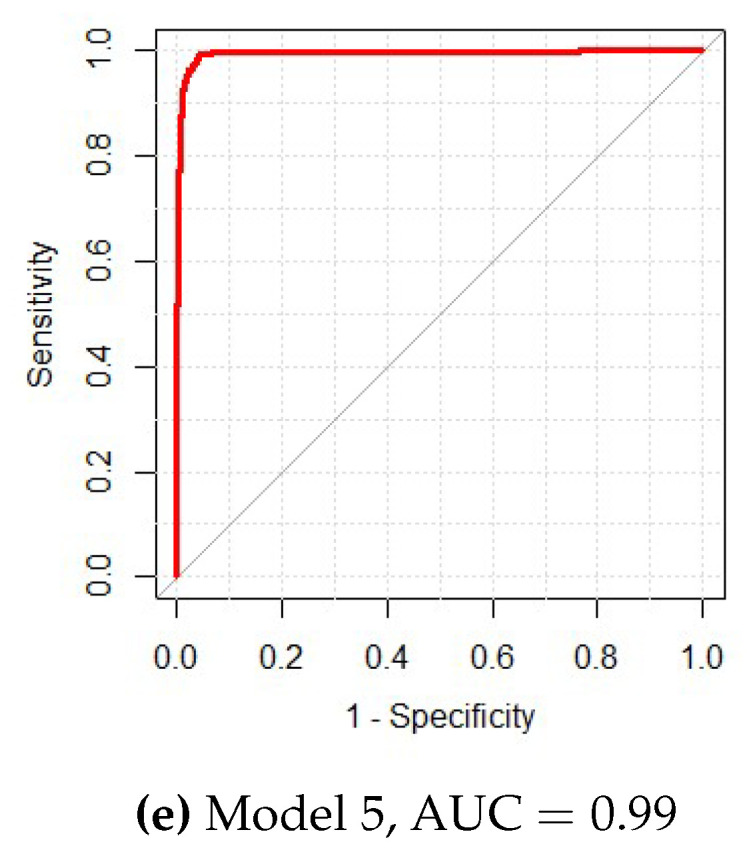
Comparisons of ROC curves for the selected models in the ESCC data set by the sequentially selected order: Model 1: −2.52+0.02×Age−1.86×Gender; Model 2: −20.64+0.08×Age−2.12×Gender+2.02×*miR-4783-3p*; Model 3: −24.21+0.09×Age−2.16×Gender+1.44×*miR-4783-3p*−1.31×*miR-320b*; Model 4: −35.70+0.10×Age−2.02×Gender+1.40×*miR-4783-3p*−0.98×*miR-320b*+1.91×*miR-1225-3p*; Model 5: −53.10+0.10×Age−1.85×Gender+1.43×*miR-4783-3p*−0.92×*miR-320b*+1.43×*miR-1225-3p*+2.10× *miR-6789-5p*.

In the testing dataset, the model had an area under the receiver operating curve (AUC) of 0.99 and achieved a high accuracy of 0.96, with a sensitivity and specificity of 0.97 and 0.95, respectively. Additionally, using the testing cohort, we evaluated the performances of the models sequentially selected by STEPWISE. Starting with a model containing age and gender, STEPWISE selected *miR-4783-3p*, *miR-320b* and *miR-1225-3p* in turn. Figure 2, showing the corresponding receiver operating curves (ROC) for these sequential models, revealed the improvement by sequentially adding predictors to the model and justified the importance of these variables in the final model. In addition, Figure 2e illustrated that adding an extra miRNA selected by FR and SC made little improvement of the model’s predictive power.

Furthermore, we conducted subgroup analysis within the testing cohort to study how the sensitivity of the final model differed by cancer stage, one of the most important risk factors. The sensitivities for stages 0, i.e., non-invasive cancer, 9 (n=27), 1 (n=128), 2 (n=57), 3 (n=61) and 4 (n=10) were 1.00, 0.98, 0.97, 0.97 and 1.00, respectively. We next evaluated how the specificity varied across controls coming from different data sources. The specificities for the various control groups, namely, NCCB (n=306), NCGG (n=2512) and MC (n=1864), were 0.99, 0.99 and 0.98, respectively. The results indicated the robust performance of the miRNA-based model toward cancer stages and data sources.

**Figure 3:**
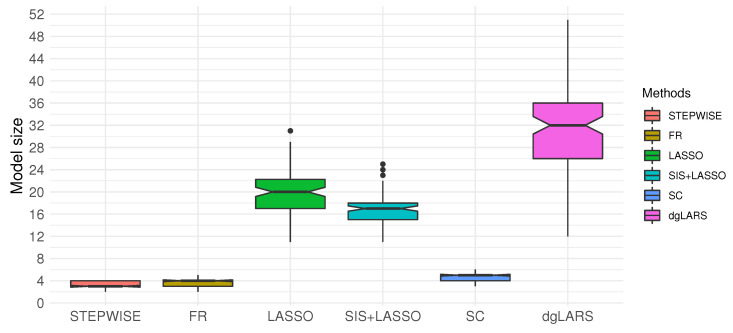
Box plot of model sizes for each method based on 100 ESCC training datasets. Performance of STEPWISE is reported with $\eta_{1}=0$ and $\eta_{2}=3.5$. Performances of SC and FR are reported with γ=0.

Finally, to compare STEPWISE with the other competing methods, we repeatedly applied the aforementioned balance sampling procedure and split the ESCC data into the training and testing sets 100 times. We obtained MSPE and the average of accuracy, sensitivity, specificity, and AUC. Figure 3 reported the model size of each method. Though STEPWISE selected fewer variables compared to the other variable selection methods (for example, LASSO selected 11-31 variables and dgLARS selected 12–51 variables), it achieved comparable prediction accuracy, specificity, sensitivity and AUC (see Table 7), evidencing the utility of STEPWISE for generating parsimonious models while maintaining competitive predictability.

We used R software [46] to obtain the numerical results in Section 4 and Section 5 with following packages: ggplot2 [47], ncvreg [32], glmnet [31], dglars [34] and screening [33].

## 6. Discussion

We have proposed to apply STEPWISE to produce final models in ultrahigh dimensional settings, without resorting to a pre-screening step. We have shown that the method identifies or includes the true model with probability going to 1, and produces consistent coefficient estimates, which are useful for properly interpreting the actual impacts of risk factors. The theoretical properties of STEPWISE were established under mild conditions, which are worth discussing. As in practice covariates are often standardized for various reasons, Condition (2) is assumed without loss of generality. Conditions (3) and (4) are generally satisfied under common GLM models, including Gaussian, binomial, Poisson and gamma distributions. Condition (5) is also often satisfied in practice. Proposition 2 in [26] may be used as a tool to verify Condition (5) as well. Conditions (1) and (6) are in good faith with the unknown true model size |M| and minimum signal strength $n^{-\alpha }$ in practice. The "irrepresentable" condition (6) is strong and may not hold in some real datasets, see, e.g., [48,49]. However, the condition holds under some commonly used covariance structures, including AR(1) and compound symmetry structure [48].

As shown in simulation studies and real data analyses, STEPWISE tends to generate models as predictive as the other well-known methods, with fewer variables (Figure 3). Parsimonious models are useful for biomedical studies as they explain data with a small number of important predictors, and offer practitioners a realistic list of biomarkers to investigate. With categorical outcome data frequently observed in biomedical studies (e.g., histology types of cancer), STEPWISE can be extended to accommodate multinomial classification, with more involved notation and computation. We will pursue this elsewhere.

There are several open questions. First, our final model was determined by using (E)BIC, which involves two extra parameters $\eta_{1}$ and $\eta_{2}$. In our numerical experiments, we used cross-validation to choose them, which seemed to work well. However, more in-depth research is needed to find their optimal values to strike a balance between false positives and false negatives. Second, despite our consistent estimates, drawing inferences based on them remains challenging. Statistical inference, which accounts for uncertainty in estimation, is key for properly interpreting analysis results and drawing appropriate conclusions. Our asymptotic results, nevertheless, are a stepping stone toward this important problem.

## Figures and Tables

**Table 1:** The values of $\eta_{1}$ and $\eta_{2}$ used in the simulation studies.

	Normal Model	Binomial Model	Poisson Model
Example 1	(0.5, 3)	(0.5, 3)	(1, 3)
Example 2	(0.5, 3)	(1, 3)	(1, 3)
Example 3	(1, 3)	(0.5, 3)	(0.5, 1)
Example 4	(1, 3.5)	(0, 1)	(1, 3)
Example 5	(0.5, 3)	(0.5, 2)	(0.5, 3)

Note: values for $\eta_{1}$ and $\eta_{2}$ were searched on the grid {0,0.25,0.5,1} and {1,2,3,3.5,4,4.5,5}, respectively.

**Table 2:** Normal model.

Example	Method	TP	FP	PIT	MSE $\left(\times 10^{-4}\right)$	MSPE
1 ($p_{0}=8$)	LASSO(1SE)	8.00 (0.00)	5.48 (6.61)	1.00 (0.00)	2.45	1.148
	LASSO(BIC)	8.00 (0.00)	2.55 (2.48)	1.00 (0.00)	2.58	1.172
	SIS+LASSO(1SE)	8.00 (0.00)	6.59 (4.22)	1.00 (0.00)	1.49	1.042
	SIS+LASSO(BIC)	8.00 (0.00)	6.04 (3.33)	1.00 (0.00)	1.37	1.025
	dgLARS(BIC)	8.00 (0.00)	3.52(2.53)	1.00 (0.00)	2.25	1.130
	SC ($\gamma_{L}$)	8.00 (0.00)	3.01 (1.85)	1.00 (0.00)	1.09	0.895
	SC ($\gamma_{H}$)	7.60 (1.59)	0.00 (0.00)	0.94 (0.24)	14.56	5.081
	FR ($\gamma_{L}$)	8.00 (0.00)	2.96 (2.04)	1.00 (0.00)	1.08	0.896
	FR ($\gamma_{H}$)	7.88 (0.84)	0.00 (0.00)	0.98 (0.14)	3.74	2.040
	STEPWISE	8.00 (0.00)	0.00 (0.00)	1.00 (0.00)	0.21	0.972
2 ($p_{0}=8$)	LASSO(1SE)	8.00 (0.00)	4.74 (4.24)	1.00 (0.00)	2.46	1.154
	LASSO(BIC)	8.00 (0.00)	2.12 (2.02)	1.00 (0.00)	2.62	1.182
	SIS+LASSO	7.99 (0.10)	6.84 (4.57)	0.99 (0.10)	1.65	1.058
	SIS+LASSO(BIC)	7.99 (0.10)	6.11 (3.85)	0.99 (0.10)	1.56	1.041
	dgLARS(BIC)	8.00 (0.00)	3.26(2.62)	1.00 (0.00)	2.28	1.138
	SC ($\gamma_{L}$)	8.00 (0.00)	2.73 (1.53)	1.00 (0.00)	0.98	0.901
	SC ($\gamma_{H}$)	7.30 (2.11)	0.00 (0.00)	0.90 (0.30)	23.70	6.397
	FR ($\gamma_{L}$)	8.00 (0.00)	2.45 (1.65)	1.00 (0.00)	0.92	0.907
	FR ($\gamma_{H}$)	7.94 (0.60)	0.00 (0.00)	0.99 (0.00)	2.69	2.062
	STEPWISE	8.00 (0.00)	0.01 (0.10)	1.00 (0.00)	0.21	0.972
3 ($p_{0}=5$)	LASSO(1SE)	5.00 (0.00)	8.24 (2.63)	1.00 (0.00)	3.07	1.084
	LASSO(BIC)	5.00 (0.00)	12.33 (3.28)	1.00 (0.00)	27.97	2.398
	SIS+LASSO(1SE)	0.97 (0.26)	15.94 (2.93)	0.00 (0.00)	1406.22	76.024
	SIS+LASSO(BIC)	0.97 (0.26)	16.20 (2.81)	0.00 (0.00)	1354.54	71.017
	dgLARS(BIC)	5.00 (0.00)	53.91 (14.44)	1.00 (0.00)	6.63	0.979
	SC ($\gamma_{L}$)	4.48 (0.50)	0.25 (0.44)	0.48 (0.50)	21.74	3.086
	SC ($\gamma_{H}$)	4.48 (0.50)	0.14 (0.35)	0.48 (0.50)	21.70	2.065
	FR ($\gamma_{L}$)	5.00 (0.00)	0.23 (0.66)	1.00 (0.00)	0.27	0.973
	FR ($\gamma_{H}$)	5.00 (0.00)	0.14 (0.35)	1.00 (0.00)	0.15	0.074
	STEPWISE	5.00 (0.00)	0.03 (0.22)	1.00 (0.00)	0.18	0.976
4 ($p_{0}=14$)	LASSO(1SE)	14.00 (0.00)	29.84 (15.25)	1.00 (0.00)	13.97	1.148
	LASSO(BIC)	13.94 (0.24)	4.92 (5.54)	0.94 (0.24)	38.69	1.995
	SIS+LASSO(1SE)	11.44 (1.45)	15.19 (7.29)	0.05 (0.21)	133.38	4.714
	SIS+LASSO(BIC)	11.35 (1.51)	10.98 (7.19)	0.05 (0.21)	137.06	4.940
	dgLARS(BIC)	14.00 (0.00)	13.93 (6.68)	1.00 (0.00)	18.08	1.329
	SC ($\gamma_{L}$)	13.68 (0.60)	0.86 (0.62)	0.75 (0.44)	11.80	1.148
	SC ($\gamma_{H}$)	4.20 (2.80)	0.03 (0.17)	0.03 (0.17)	407.86	6.567
	FR ($\gamma_{L}$)	14.00 (0.00)	0.50 (0.76)	1.00 (0.00)	1.23	0.940
	FR ($\gamma_{H}$)	4.99 (3.07)	0.00 (0.00)	0.03 (0.17)	360.65	6.640
	STEPWISE	14.00 (0.00)	0.00 (0.00)	1.00 (0.00)	0.91	0.958
5 ($p_{0}=3$)	LASSO(1SE)	3.00 (0.00)	22.76 (9.05)	1.00 (0.00)	1.01	0.044
	LASSO(BIC)	3.00 (0.00)	8.29 (3.23)	1.00 (0.00)	1.75	0.054
	SIS+LASSO(1SE)	3.00 (0.00)	8.40 (3.10)	1.00 (0.00)	0.44	0.041
	SIS+LASSO(BIC)	3.00 (0.00)	9.58 (3.36)	1.00 (0.00)	0.29	0.040
	dgLARS(BIC)	3.00 (0.00)	13.39 (4.94)	1.00 (0.00)	1.28	0.048
	SC ($\gamma_{L}$)	3.00 (0.00)	1.47 (0.67)	1.00 (0.00)	0.03	0.038
	SC ($\gamma_{H}$)	2.01 (0.10)	0.01 (0.10)	0.01 (0.10)	4.51	0.008
	FR ($\gamma_{L}$)	3.00 (0.00)	1.21 (1.01)	1.00 (0.00)	0.03	0.038
	FR ( $\gamma_{H}$)	3.00 (0.00)	0.00 (0.00)	1.00 (0.00)	0.01	0.003
	STEPWISE	3.00 (0.00)	0.00 (0.00)	1.00 (0.00)	0.01	0.039

Note: TP, true positives; FP, false positives; PIT, probability of including all true predictors in the selected predictors; MSE, mean squared error of $\hat{\beta }$; MSPE, mean squared prediction error; numbers in the parentheses are standard deviations; LASSO(BIC), LASSO with the tuning parameter chosen to give the smallest BIC for the models on the LASSO path; LASSO(1SE), LASSO with the tuning parameter chosen by the one-standard-error rule; SIS+LASSO(BIC), sure independence screening by [5] followed by LASSO(BIC); SIS+LASSO(1SE), sure independence screening followed by LASSO(1SE); dgLARS(BIC), differential geometric least angle regression by [11,12] with the tuning parameter chosen to give the smallest BIC on the dgLARS path; SC(γ), sequentially conditioning approach by [8]; FR(γ), forward regression by [7]; STEPWISE, the proposed method; in FR and SC, the smaller and large values of γ are presented as $\gamma_{L}$ and $\gamma_{H}$, respectively; $p_{0}$ denotes the number of true signals; LASSO(1SE), LASSO(BIC), SIS and dgLARS were conducted via R packages glmnet [31], ncvreg [32], screening [33] and dglars [34], respectively.

**Table 3:** Binomial model.

Example	Method	TP	FP	PIT	MSE	MSPE
1 ($p_{0}=8$)	LASSO(1SE)	7.99 (0.10)	4.77 (5.56)	0.99 (0.10)	0.021	0.104
	LASSO(BIC)	7.99 (0.10)	3.19 (2.34)	0.99 (0.10)	0.021	0.104
	SIS+LASSO(1SE)	7.94 (0.24)	35.42 (6.77)	0.94 (0.24)	0.119	0.048
	SIS+LASSO(BIC)	7.94 (0.24)	16.83 (21.60)	0.94 (0.24)	0.119	0.073
	dgLARS(BIC)	8.00 (0.00)	3.27 (2.29)	1.00 (0.00)	0.019	0.102
	SC ($\gamma_{L}$)	8.00 (0.00)	2.81 (1.47)	1.00 (0.00)	0.009	0.073
	SC ($\gamma_{H}$)	1.02 (0.14)	0.00 (0.00)	0.00 (0.00)	0.030	0.028
	FR ($\gamma_{L}$)	8.00 (0.00)	3.90 (2.36)	1.00 (0.00)	0.032	0.066
	FR ($\gamma_{H}$)	2.00 (0.00)	0.00 (0.00)	0.00 (0.00)	0.025	0.027
	STEPWISE	7.98 (0.14)	0.08 (0.53)	0.98 (0.14)	0.002	0.094
2 ($p_{0}=8$)	LASSO(1SE)	7.98 (0.14)	3.29 (2.76)	0.98 (0.14)	0.054	0.073
	LASSO(BIC)	7.99 (0.10)	3.84 (2.72)	0.99 (0.10)	0.052	0.067
	SIS+LASSO(1SE)	7.92 (0.27)	28.20 (7.31)	0.92 (0.27)	0.038	0.030
	SIS+LASSO(BIC)	7.92 (0.27)	9.60 (12.92)	0.92 (0.27)	0.051	0.058
	dgLARS(BIC)	7.99 (0.10)	3.94 (2.65)	0.99 (0.10)	0.050	0.067
	SC ($\gamma_{L}$)	7.72 (0.45)	0.39 (0.49)	0.72 (0.45)	0.005	0.063
	SC ($\gamma_{H}$)	1.13 (0.37)	0.00 (0.00)	0.00 (0.00)	0.069	0.044
	FR ($\gamma_{L}$)	7.99 (0.10)	0.66 (0.76)	0.99 (0.10)	0.014	0.051
	FR ($\gamma_{H}$)	2.10 (0.30)	0.00 (0.00)	0.00 (0.00)	0.061	0.033
	STEPWISE	7.99 (0.10)	0.02 (0.14)	0.99 (0.10)	0.004	0.056
3 ($p_{0}=5$)	LASSO(1SE)	4.51 (0.52)	7.36 (2.57)	0.52 (0.50)	0.155	0.051
	LASSO(BIC)	4.98 (0.14)	5.97 (2.25)	0.98 (0.14)	0.118	0.037
	SIS+LASSO(1SE)	0.85 (0.46)	10.66 (3.01)	0.00 (0.00)	0.206	0.186
	SIS+LASSO(BIC)	0.85 (0.46)	12.10 (3.13)	0.00 (0.00)	0.197	0.185
	dgLARS(BIC)	4.92 (0.27)	16.21 (6.21)	0.92 (0.27)	0.112	0.035
	SC ($\gamma_{L}$)	4.32 (0.49)	0.47 (0.50)	0.33 (0.47)	0.016	0.048
	SC ($\gamma_{H}$)	2.62 (1.34)	0.42 (0.50)	0.00 (0.00)	0.104	0.066
	FR ($\gamma_{L}$)	4.98 (0.14)	0.67 (0.79)	0.98 (0.14)	0.020	0.033
	FR ($\gamma_{H}$)	2.98 (0.95)	0.40 (0.49)	0.00 (0.00)	0.087	0.043
	STEPWISE	4.97 (0.17)	0.04 (0.28)	0.97 (0.17)	0.014	0.034
4 ($p_{0}=14$)	LASSO(1SE)	9.96 (1.89)	6.78 (7.92)	0.01 (0.01)	0.112	0.107
	LASSO(BIC)	9.33 (1.86)	2.79 (2.87)	0.00 (0.00)	0.112	0.118
	SIS+LASSO(1SE)	10.03 (1.62)	28.01 (9.54)	0.03 (0.17)	0.098	0.070
	SIS+LASSO(BIC)	8.90 (1.99)	5.42 (10.64)	0.01 (0.10)	0.114	0.120
	dgLARS(BIC)	9.31 (1.85)	2.84 (2.86)	0.00 (0.00)	0.110	0.117
	SC ($\gamma_{L}$)	9.48 (1.40)	2.35 (2.14)	0.00 (0.00)	0.043	0.070
	SC ($\gamma_{H}$)	1.17 (0.40)	0.00 (0.00)	0.00 (0.00)	0.125	0.049
	FR ($\gamma_{L}$)	11.83 (1.39)	1.58 (1.60)	0.09 (0.29)	0.026	0.048
	FR ($\gamma_{H}$)	2.06 (0.24)	0.00 (0.00)	0.00 (0.00)	0.119	0.032
	STEPWISE	11.81 (1.42)	1.52 (1.58)	0.09 (0.29)	0.026	0.048
5 ($p_{0}=3$)	LASSO(1SE)	2.00 (0.00)	1.55 (1.76)	0.00 (0.00)	0.008	0.215
	LASSO(BIC)	2.00 (0.00)	1.86 (1.57)	0.00 (0.00)	0.008	0.213
	SIS+LASSO(1SE)	2.23 (0.42)	10.81 (6.45)	0.23 (0.42)	0.007	0.192
	SIS+LASSO(BIC)	2.10 (0.30)	3.60 (4.65)	0.10 (0.30)	0.007	0.206
	dgLARS(BIC)	2.00 (0.00)	1.64 (1.49)	0.00 (0.00)	0.008	0.213
	SC ($\gamma_{L}$)	2.27 (0.49)	7.16 (3.20)	0.29 (0.46)	0.060	0.166
	SC ($\gamma_{H}$)	1.87 (0.34)	0.03 (0.17)	0.00 (0.00)	0.005	0.030
	FR ($\gamma_{L}$)	2.96 (0.20)	8.88 (5.39)	0.96 (0.20)	0.013	0.147
	FR ( $\gamma_{H}$)	1.97 (0.17)	0.03 (0.17)	0.00 (0.00)	0.005	0.019
	STEPWISE	2.89 (0.31)	0.76 (1.70)	0.89 (0.31)	0.001	0.194

Note: abbreviations are explained in the footnote of Table 2.

**Table 4:** Poisson model.

Example	Method	TP	FP	PIT	MSE	MSPE
1 ($p_{0}=8$)	LASSO(1SE)	7.93 (0.43)	4.64 (4.82)	0.96 (0.19)	0.001	4.236
	LASSO(BIC)	7.99 (0.10)	14.37 (14.54)	0.99 (0.10)	0.001	3.133
	SIS+LASSO(1SE)	7.89 (0.37)	25.37 (8.39)	0.91 (0.29)	0.001	3.247
	SIS+LASSO(BIC)	7.89 (0.37)	17.77 (11.70)	0.91 (0.29)	0.001	3.078
	dgLARS(BIC)	8.00 (0.00)	13.28 (14.31)	1.00 (0.00)	0.001	3.183
	SC ($\gamma_{L}$)	7.96 (0.20)	4.94 (3.46)	0.96 (0.20)	0.001	2.874
	SC ($\gamma_{H}$)	5.05 (1.70)	0.04 (0.24)	0.07 (0.26)	0.001	3.902
	FR ($\gamma_{L}$)	7.93 (0.26)	4.86 (3.73)	0.93 (0.26)	0.001	2.837
	FR ($\gamma_{H}$)	5.13 (1.61)	0.06 (0.31)	0.07 (0.26)	0.001	3.833
	STEPWISE	7.91 (0.29)	2.77 (2.91)	0.91 (0.29)	0.001	3.410
2 ($p_{0}=8$)	LASSO(1SE)	8.00 (0.00)	2.23 (3.52)	1.00 (0.00)	0.001	3.981
	LASSO(BIC)	8.00 (0.00)	8.98 (8.92)	1.00 (0.00)	0.001	3.107
	SIS+LASSO(1SE)	7.98 (0.14)	22.85 (7.08)	0.98 (0.14)	0.001	2.824
	SIS+LASSO(BIC)	7.98 (0.14)	13.55 (8.24)	0.98 (0.14)	0.001	2.937
	dgLARS(BIC)	8.00 (0.00)	8.91 (9.10)	1.00 (0.00)	0.001	3.099
	SC ($\gamma_{L}$)	8.00 (0.00)	3.89 (2.89)	1.00 (0.00)	0.000	2.979
	SC ($\gamma_{H}$)	5.68 (1.45)	0.00 (0.00)	0.12 (0.33)	0.001	3.971
	FR ($\gamma_{L}$)	8.00 (0.00)	3.60 (2.80)	1.00 (0.00)	0.000	3.032
	FR ($\gamma_{H}$)	5.71 (1.42)	0.00 (0.00)	0.10 (0.30)	0.001	3.911
	STEPWISE	7.98 (0.14)	2.00 (2.23)	0.98 (0.14)	0.000	3.589
3 ($p_{0}=5$)	LASSO(1SE)	4.37 (0.51)	6.88 (2.61)	0.38(0.48)	0.001	1.959
	LASSO(BIC)	4.79 (0.41)	5.62 (2.17)	0.79 (0.41)	0.000	2.044
	SIS+LASSO(1SE)	0.86 (0.47)	10.11 (2.55)	0.00 (0.00)	0.002	3.266
	SIS+LASSO(BIC)	0.86 (0.47)	11.86 (2.99)	0.00 (0.00)	0.002	3.160
	dgLARS(BIC)	4.55 (0.51)	18.29 (6.13)	0.56 (0.49)	0.001	1.877
	SC ($\gamma_{L}$)	4.73 (0.45)	0.53 (0.66)	0.73 (0.45)	0.000	2.479
	SC ($\gamma_{H}$)	2.84 (0.63)	0.40 (0.49)	0.00 (0.00)	0.001	0.664
	FR ($\gamma_{L}$)	4.54 (0.52)	1.98 (2.19)	0.55 (0.50)	0.000	2.128
	FR ($\gamma_{H}$)	2.71 (0.70)	0.43 (0.50)	0.00 (0.00)	0.001	0.605
	STEPWISE	4.54 (0.52)	1.77 (2.01)	0.55 (0.50)	0.000	2.132
4 ($p_{0}=14$)	LASSO(1SE)	10.01 (1.73)	3.91 (6.03)	0.01 (0.10)	0.003	15.582
	LASSO(BIC)	12.11 (1.46)	36.56 (22.43)	0.19 (0.39)	0.002	5.688
	SIS+LASSO(1SE)	10.42 (1.66)	21.41 (8.87)	0.03 (0.17)	0.003	11.316
	SIS+LASSO(BIC)	10.73 (1.66)	32.67 (8.92)	0.03 (0.17)	0.003	8.545
	dgLARS(BIC)	12.05 (1.52)	38.70 (28.97)	0.18 (0.38)	0.002	5.111
	SC ($\gamma_{L}$)	10.33 (1.63)	10.48 (6.66)	0.02 (0.14)	0.002	4.499
	SC ($\gamma_{H}$)	5.32 (1.92)	0.52 (1.37)	0.00 (0.00)	0.003	14.005
	FR ($\gamma_{L}$)	12.00 (1.71)	8.93 (6.36)	0.23 (0.42)	0.001	4.503
	FR ($\gamma_{H}$)	5.65 (2.13)	0.38 (1.15)	0.00 (0.00)	0.003	13.802
	STEPWISE	11.80 (1.72)	5.97 (5.37)	0.19 (0.39)	0.001	5.809
5 ($p_{0}=3$)	LASSO(1SE)	2.00 (0.00)	1.13 (2.85)	0.00 (0.00)	0.003	2.674
	LASSO(BIC)	2.01 (0.10)	2.82 (2.52)	0.01 (0.10)	0.003	2.583
	SIS+LASSO(1SE)	2.87 (0.34)	9.28 (3.85)	0.87 (0.34)	0.002	2.455
	SIS+LASSO(BIC)	2.87 (0.34)	9.88 (4.29)	0.87 (0.34)	0.002	2.355
	dgLARS(BIC)	2.00 (0.00)	2.88 (2.38)	0.00 (0.00)	0.003	2.562
	SC ($\gamma_{L}$)	2.75 (0.44)	3.27 (1.75)	0.75 (0.44)	0.001	2.339
	SC ($\gamma_{H}$)	2.00 (0.00)	0.00 (0.00)	0.00 (0.00)	0.003	1.086
	FR ($\gamma_{L}$)	3.00 (0.00)	2.80 (1.73)	1.00 (0.00)	0.001	2.326
	FR ($\gamma_{H}$)	2.40 (0.49)	0.00 (0.00)	0.40 (0.49)	0.002	0.981
	STEPWISE	3.00 (0.00)	0.35 (0.59)	1.00 (0.00)	0.001	2.977

Note: abbreviations are explained in the footnote of Table 2.

**Table 5:** Comparisons of MSPE among competing methods using the mammalian eye data set.

	STEPWISE	FR	LASSO	SIS+LASSO	SC	dgLARS
Training set	0.005	0.005	0.005	0.006	0.005	0.014
Testing set	0.011	0.012	0.010	0.009	0.014	0.020

Note: The mean squared prediction error (MSPE) was averaged over 120 splits. LASSO, least absolute shrinkage and selection operator with regularization parameter that gives the smallest BIC; SIS+LASSO, sure independence screening by [5] followed by LASSO; dgLARS, differential geometric least angle regression by [11,12] that gives the smallest BIC; SC(γ), sequentially conditioning approach by [8]; FR(γ), forward regression by [7]; STEPWISE, the proposed method. STEPWISE was performed with $\eta_{1}=1$ and $\eta_{2}=4$; FR and SC were performed with γ=1.

**Table 6:** Clinicopathological characteristics of study participants of the ESCC data set.

Covariates	Training Set	Testing set
	$n_{1}$ (%)	$n_{2}$ (%)
**Esophageal squamous cell carcinoma (ESCC) patients**
Total number of patients	283	283
Age, median (range)	65 [40, 86]	67 [37, 90]
Gender:
Male	224 (79.0%)	247 (87.3%)
Female	59 (21.0%)	36 (12.7%)
Stage:
0	24 (8.5%)	27 (9.5%)
1	127 (44.9%)	128 (45.2%)
2	58 (20.5%)	57 (20.1%)
3	67 (23.7%)	61 (21.6%)
4	7 (2.4%)	10 (3.6%)
**Non-ESCC Controls**
Total number of patients	283	4,682
Age, median (range)	68 [27, 92]	67.5 [20, 100]
Gender:
Male	131 (46.3%)	2,086 (44.5%)
Female	152 (53.7%)	2,596 (55.5%)
Data sources of the controls:
National Cancer Center Biobank (NCCB)	17 (6.0%)	306 (6.5%)
National Center for Geriatrics and Gerontology (NCGG)	158 (55.8%)	2,512 (53.7%)
Minoru clinic (MC)	108 (38.2%)	1,864 (39.8%)

**Table 7:** Comparisons of competing methods over 100 independent splits of the ESCC data into training and testing sets.

Training Set	MSPE	Accuracy	Sensitivity	Specificity	AUC
STEPWISE	0.02	0.97	0.98	0.97	1.00
SC	0.01	0.99	0.98	0.98	1.00
FR	0.02	0.99	0.97	0.97	1.00
LASSO	0.01	0.98	1.00	0.97	1.00
SIS+LASSO	0.01	0.99	1.00	0.99	1.00
dgLARS	0.04	0.96	0.99	0.94	1.00
**Training Set**	**MSPE**	**Accuracy**	**Sensitivity**	**Specificity**	**AUC**
STEPWISE	0.04	0.96	0.97	0.95	0.99
SC	0.03	0.96	0.97	0.96	0.99
FR	0.04	0.96	0.97	0.95	0.99
LASSO	0.03	0.96	0.99	0.95	1.00
SIS+LASSO	0.02	0.97	0.99	0.96	1.00
dgLARS	0.05	0.94	0.98	0.94	1.00

Note: Values were averaged over 100 splits. STEPWISE was performed with $\eta_{1}=0$ and $\eta_{2}=1$. SC and FR were performed with γ=1. The regularization parameters in LASSO and dgLARS were selected to minimize the BIC.

## Supplementary Materials

An R package, STEPWISE, was developed and is available at https://github.com/AlexPijyan/STEPWISE, along with the examples shown in the paper.

## Appendix A. Proofs of Main Theorems

Since b(·) is twice continuously differentiable with a nonnegative second derivative $b^{\prime \prime }\left(\;\cdot \;\right)$, $b_{\max}:=\max_{\left|t\right|\leq K^{3}}\left|b\left(t\right)\right|$, $\mu_{\max}:=\max_{\left|t\right|\leq K^{3}}\left|b^{\prime }\left(t\right)\right|$ and $\sigma_{\max}:=\sup_{\left|t\right|\leq K^{3}}\left|b^{\prime \prime }\left(t\right)\right|$ are bounded above, where *L* and *K* are some constants from Conditions (1) and (2), respectively. Let $G_{n}\left\{f\left(\xi \right)\right\}=n^{-1/2}\sum_{i=1}^{n}\left(f\left(\xi_{i}\right)-E\left[f\left(\xi_{i}\right)\right]\right)$ for a sequence of i.i.d. random variables $\xi_{i}$(i=1,…,n) and a non-random function f(·).

Given any $\beta_{S}$, when a variable $X_{r},r\in S^{c}$ is added into the model *S*, we define the augmented log-likelihood as

(A1) $$\ell_{S\cup \{r\}}\left(\beta_{S+r}\right):=E_{n}\left\{L\left(\beta_{S}^{T}X_{S}+\beta_{r}X_{r},Y\right)\right\}.$$

We use ${\hat{\beta }}_{S+r}$ to denote the maximizer of (A1). Thus, ${\hat{\beta }}_{S+r}={\hat{\beta }}_{S\cup \{r\}}$. In addition, denote the maximizer of $E[\ell_{S\cup \{r\}}\left(\beta_{S+r}\right)]$ by $\beta_{S+r}^{*}$. Due to the concavity of the log-likelihood in GLMs with the canonical link, $\beta_{S+r}^{*}$ is unique.

**Proof** **of** **Theorem 1.** Given an index set *S* and $r\in S^{c}$, let $B_{S}^{0}\left(d\right)=\{\beta_{S}:∥\beta_{S}-\beta_{S}^{*}∥\leq d/\left(K\sqrt{\left|S\right|}\right)\}$ where $d=A_{2}\sqrt{q^{3}\log p/n}$ with $A_{2}$ defined in Lemma A6. Let Ω be the event that

$$\begin{array}{cc} \{ & \underset{\left|S\right|\leq q,\beta_{S}\in B_{S}^{0}\left(d\right)}{\sup}\left|G_{n}\left[L\left(\beta_{S}^{T}X_{S},Y\right)-L\left(\beta_{S}^{*T}X_{S},Y\right)\right]\right|\leq 20A_{1}d\sqrt{q\log p}\;\;\mathrm{and}\;\\ \; & \max_{|S|\leq q}\left|G_{n}\left[L(\beta_{S}^{*T}X_{S},Y)\right]\right|\leq 10\left(A_{1}K^{2}+b_{\max}\right)\sqrt{q\log p}\}, \end{array}$$

where $A_{1}$ is some constant defined in Lemma A4. By Lemma A4, P(Ω)≥1−6exp(−6qlogp). Thus in the rest of the proof, we only consider the sample points in Ω. In the proof of Lemma A6, we show that $\max_{|S|\leq q}∥{\hat{\beta }}_{S}-\beta_{S}^{*}∥\leq A_{2}K^{-1}{\left(q^{2}\log p/n\right)}^{1/2}$ under Ω. Then given an index set *S* and $\beta_{S}$ such that $|S|<q,∥\beta_{S}-\beta_{S}^{*}∥\leq A_{2}K^{-1}{\left(q^{2}\log p/n\right)}^{1/2}$, and for any $j\in S^{c}$,

$$\begin{array}{cc} \; & \;\ell_{S\cup \{j\}}\left(\beta_{S+j}^{*}\right)-\ell_{S}\left({\hat{\beta }}_{S}\right)\geq \underset{∥\beta_{S}-\beta_{S}^{*}∥\leq A_{2}K^{-1}{\left(q^{2}\log p/n\right)}^{1/2}}{\inf}\ell_{S\cup \{j\}}\left(\beta_{S+j}^{*}\right)-\ell_{S}\left(\beta_{S}\right)\\ \; & =n^{-1/2}G_{n}\left[L(\beta_{S+j}^{*T}X_{S\cup \{j\}},Y)\right]-n^{-1/2}G_{n}\left[L(\beta_{S}^{*T}X_{S},Y)\right]\\ \; & \;-\underset{∥\beta_{S}-\beta_{S}^{*}∥\leq A_{2}K^{-1}{\left(q^{2}\log p/n\right)}^{1/2}}{\sup}\left|n^{-1/2}G_{n}\left[L\left(\beta_{S}^{T}X_{S},Y\right)-L\left(\beta_{S}^{*T}X_{S},Y\right)\right]\right|\\ \; & \;+E\left[L(\beta_{S+j}^{*T}X_{S\cup \{j\}},Y)\right]-E\left[L(\beta_{S}^{*T}X_{S},Y)\right]\\ \; & \geq -20\left(A_{1}K^{2}+b_{\max}\right)\sqrt{q\log p/n}-20A_{1}A_{2}q^{2}\log p/n+\frac{\sigma_{\min}\kappa_{\min}}{2}{∥\beta_{S+j}^{*}-{\left(\beta_{S}^{*T},0\right)}^{T}∥}^{2}, \end{array}$$

where the second inequality follows from the event Ω and Lemma A5. By Lemma A1, if M⊈S, there exists $r\in S^{c}\cap M$, such that $∥\beta_{S+r}^{*T}-\left(\beta_{S}^{*T},0\right)∥\geq C\sigma_{\max}^{-1}\kappa_{\max}^{-1}n^{-\alpha }$. Thus, there exists some constant $C_{1}$ that does not depend on *n* such that

(A2) $$\begin{array}{ccc} & & \max_{j\in S^{c}}\ell_{S\cup \{j\}}\left({\hat{\beta }}_{S+j}\right)-\ell_{S}\left({\hat{\beta }}_{S}\right)\geq \max_{j\in S^{c}}\ell_{S\cup \{j\}}\left(\beta_{S+j}^{*}\right)-\ell_{S}\left({\hat{\beta }}_{S}\right)\geq \ell_{S\cup \{r\}}\left(\beta_{S+r}^{*}\right)-\ell_{S}\left({\hat{\beta }}_{S}\right)\\ & & \geq -20\left(A_{1}K^{2}+b_{\max}\right)\sqrt{q\log p/n}-20A_{1}A_{2}q^{2}\log p/n+\frac{C^{2}\sigma_{\min}\kappa_{\min}n^{-2\alpha }}{2\sigma_{\max}^{2}\kappa_{\max}^{2}}\geq C_{1}n^{-2\alpha }, \end{array}$$

where the first inequality follows from ${\hat{\beta }}_{S+j}$ being the maximizer of (A1) and the second inequality follows from Conditions (1) and (6). Withdrawing the restriction to Ω, we obtain that

$$\begin{array}{c} P\left(\min_{|S|<q,M⊈S}\max_{j\in S^{c}}\ell_{S\cup \{j\}}\left({\hat{\beta }}_{S\cup \{j\}}\right)-\ell_{S}\left({\hat{\beta }}_{S}\right)\geq C_{1}n^{-2\alpha }\right)\geq 1-6\exp\left(-6q\log p\right). \end{array}$$

□

**Proof** **of** **Theorem 2.** We have shown that our forward stage will not stop when M⊈Sand|S|<q with probability converging to 1. For any $r\in S^{c}\cap M^{c}$, $\beta_{S+r}^{*}$ is the unique solution to the equation $E\left[\left\{Y-\mu \left(\beta_{S+r}^{T}X_{S\cup \{r\}}\right)\right\}X_{S\cup \{r\}}\right]=0$. By the mean value theorem,

$$\begin{array}{cc} \; & E\left[\left\{Y-\mu \left(\beta_{S}^{*T}X_{S}\right)\right\}X_{r}\right]=E\left[\left\{\mu \left(\beta_{*}^{T}X\right)-\mu \left(\beta_{S}^{*T}X_{S}\right)\right\}X_{r}\right]\\ \; & =E\left[\left\{\mu \left(\beta_{*}^{T}X\right)-\mu \left(\beta_{S}^{*T}X_{S}\right)\right\}X_{r}\right]-E\left[\left\{\mu \left(\beta_{*}^{T}X\right)-\mu \left(\beta_{S+r}^{*T}X_{S\cup \{r\}}\right)\right\}X_{r}\right]\\ \; & =\left(\beta_{S+r}^{*T}-\left(\beta_{S}^{*T},0\right)\right)E\left[\sigma \left({\tilde{\beta }}_{S+r}^{T}X_{S\cup \{r\}}\right)X_{S\cup \{r\}}^{⊗2}\right]e_{r}, \end{array}$$

where ${\tilde{\beta }}_{S+r}$ is some point between $\beta_{S+r}$ and ${\left(\beta_{S}^{*T},0\right)}^{T}$ and $e_{r}$ is a vector of length (|S|+1) with the *r*th element being 1. Since $|{\tilde{\beta }}_{S+r}^{T}X_{S\cup \{r\}}\left|\leq \right|\beta_{S+r}^{*T}X_{S\cup \{r\}}\left|+\right|\left(\beta_{S}^{*T},0\right)X_{S\cup \{r\}}|\leq 2K^{2}$ by Conditions (1) and (2), $|\sigma \left({\tilde{\beta }}_{S+r}^{T}X_{S\cup \{r\}}\right)|\geq \sigma_{\min}$ and

$$\begin{array}{c} o\left(n^{-\alpha }\right)=\left|E\left[\left\{Y-\mu \left(\beta_{S}^{*T}X_{S}\right)\right\}X_{r}\right]\right|\geq \sigma_{\min}\kappa_{\min}∥\beta_{S+r}^{*T}-\left(\beta_{S}^{*T},0\right)∥. \end{array}$$

Therefore, $\max_{S:|S|\leq q,r\in S^{c}\cap M^{c}}∥\beta_{S+r}^{*T}-\left(\beta_{S}^{*T},0\right)∥=o\left(n^{-\alpha }\right)$. Under Ω that is defined in Theorem 1, $\max_{|S|\leq q}∥{\hat{\beta }}_{S}-\beta_{S}^{*}∥\leq A_{2}K^{-1}{\left(q^{2}\log p/n\right)}^{1/2}$. For any $j\in S^{c}$,

$$\begin{array}{cc} \; & \;\ell_{S\cup \{j\}}\left(\beta_{S+j}^{*}\right)-\ell_{S}\left({\hat{\beta }}_{S}\right)\leq \underset{∥\beta_{S}-\beta_{S}^{*}∥\leq A_{2}K^{-1}{\left(q^{2}\log p/n\right)}^{1/2}}{\sup}\ell_{S\cup \{j\}}\left(\beta_{S+j}^{*}\right)-\ell_{S}\left(\beta_{S}\right)\\ \; & \leq \left|n^{-1/2}G_{n}\left[L(\beta_{S+j}^{*T}X_{S\cup \{j\}},Y)\right]\right|+\left|n^{-1/2}G_{n}\left[L(\beta_{S}^{*T}X_{S},Y)\right]\right|\\ \; & \;+\underset{∥\beta_{S}-\beta_{S}^{*}∥\leq A_{2}K^{-1}{\left(q^{2}\log p/n\right)}^{1/2}}{\sup}\left|n^{-1/2}G_{n}\left[L\left(\beta_{S}^{T}X_{S},Y\right)-L\left(\beta_{S}^{*T}X_{S},Y\right)\right]\right|\\ \; & \;+\left|E\left[L(\beta_{S+j}^{*T}X_{S\cup \{j\}},Y)\right]-E\left[L(\beta_{S}^{*T}X_{S},Y)\right]\right|\\ \; & \leq 20\left(A_{1}K^{2}+b_{\max}\right)\sqrt{qn^{-1}\log p}+20A_{1}A_{2}q^{2}n^{-1}\log p+\sigma_{\max}\kappa_{\max}{∥\beta_{S+j}^{*}-{\left(\beta_{S}^{*T},0\right)}^{T}∥}^{2}/2, \end{array}$$

where the second inequality follows from the event Ω and Lemma A5. Since $\max_{S:|S|<q,r\in S^{c}\cap M^{c}}∥\beta_{S+r}^{*}-{\left(\beta_{S}^{*T},0\right)}^{T}∥=o\left(n^{-\alpha }\right)$ and $qn^{-1+4\alpha }\log p\to 0$,

$$\begin{array}{cc} \max_{S:|S|<q,r\in S^{c}\cap M^{c}}\ell_{S\cup \{r\}}\left(\beta_{S+r}^{*}\right)-\ell_{S}\left({\hat{\beta }}_{S}\right)\; & \leq 20\left(A_{1}K^{2}+b_{\max}\right)\sqrt{qn^{-1}\log p}+20A_{1}A_{2}q^{2}n^{-1}\log p\\ \; & \;+\sigma_{\max}\kappa_{\max}{∥\beta_{S+j}^{*}-{\left(\beta_{S}^{*T},0\right)}^{T}∥}^{2}/2=o\left(n^{-2\alpha }\right), \end{array}$$

with probability at least 1−6exp(−6qlogp). Then by Lemma A6,

(A3) $$\begin{array}{ccc} & & \;\max_{S:|S|<q,r\in S^{c}\cap M^{c}}\ell_{S\cup \{r\}}\left({\hat{\beta }}_{S+r}\right)-\ell_{S}\left({\hat{\beta }}_{S}\right)\\ & & \leq \max_{S:|S|<q,r\in S^{c}\cap M^{c}}\left|\ell_{S\cup \{r\}}\left({\hat{\beta }}_{S+r}\right)-\ell_{S\cup \{r\}}\left(\beta_{S+r}^{*}\right)\right|+\max_{S:|S|<q,r\in S^{c}\cap M^{c}}\left|\ell_{S\cup \{r\}}\left(\beta_{S+r}^{*}\right)-\ell_{S}\left({\hat{\beta }}_{S}\right)\right|\\ & & \leq A_{3}q^{2}n^{-1}\log p+o\left(n^{-2\alpha }\right)=o\left(n^{-2\alpha }\right), \end{array}$$

with probability at least 1−12exp(−6qlogp). By Theorem 1, if M⊈S, the forward stage would select a noise variable with probability less than 18exp(−6qlogp). For k>|M|, $M⊈S_{k}$ implies that at least k−|M| noise variables are selected within the *k* steps. Then for $k=C_{2}\left|M\right|$ with $C_{2}>2$,

PM⊈Sk≤∑j=k−|M|kkj18exp(−6qlogp)j≤|M|k|M|18exp(−6qlogp)k−|M|≤18exp(−6qlogp+log|M|+|M|logk)≤18exp(−4qlogp).

Therefore, $M\subset S_{C_{2}\left|M\right|}$ with probability at least 1−18exp(−4qlogp). □

**Proof** **of** **Theorem 3.** By Theorem 2, M will be included in $F_{k}$ for some k<q with probability going to 1. Therefore, the forward stage stops at the *k*th step if $\mathrm{EBIC}\left(F_{k+1}\right)>\mathrm{EBIC}\left(F_{k}\right)$. On the other hand, that $\mathrm{EBIC}\left(F_{k+1}\right)<\mathrm{EBIC}\left(F_{k}\right)$ if and only if $2\ell_{F_{k+1}}\left({\hat{\beta }}_{F_{k+1}}\right)-2\ell_{F_{k}}\left({\hat{\beta }}_{F_{k}}\right)\geq \left(\log n+2\eta_{1}\log p\right)/n$. Thus, to show the forward stage stops at the *k*th step, we only need to show that with probability tending to 1,

(A4) $$2\ell_{F_{k+1}}\left({\hat{\beta }}_{F_{k+1}}\right)-2\ell_{F_{k}}\left({\hat{\beta }}_{F_{k}}\right)<\left(\log n+2\eta_{1}\log p\right)/n,$$

for all $\eta_{1}>0$. To prove (A4), we first verify the conditions (A4) and (A5) in [17]. Given any index *S* such that M⊆S and |S|≤q, let $\beta_{*S}$ be the subvector of $\beta_{*}$ corresponding to *S*. We obtain that

$$\begin{array}{c} E\left[\left(Y-\mu \left(\beta_{*S}^{T}X_{S}\right)\right)X_{S}\right]=E\left[E\left[\left(Y-\mu \left(\beta_{*M}^{T}X_{M}\right)\right)|X_{S}\right]X_{S}\right]=0. \end{array}$$

This implies $\beta_{S}^{*}=\beta_{*S}$. Given any $\pi \in R^{\left|S\right|}$, let $H_{S}:=\left\{h\left(\pi ,\beta_{S}\right)=(\sigma_{\max}K^{2}{\left|S|\right)}^{-1}\sigma \left(\beta_{S}^{T}X_{S}\right){\left(\pi^{T}X_{S}\right)}^{2},∥\pi ∥=1,\beta_{S}\in B_{S}^{0}\left(d\right)\right\}$. By Conditions (1) and (2), $h(\pi ,\beta_{S})$ is bounded between −1 and 1 uniformly over ∥π∥=1 and $\beta_{S}\in B_{S}^{0}\left(d\right)$. By Lemma 2.6.15 in [50], the VC indices of $W:=\{{\left(K\sqrt{\left|S\right|}\right)}^{-1}\pi^{T}X_{S},∥\pi ∥=1\}$ and $V:=\{\beta_{S}^{T}X_{S},\beta_{S}\in B_{S}^{0}\left(d\right)\}$ are bounded by |S|+2. For the definitions of the VC index and covering numbers, we refer to pages 83 and 85 in [50]. The VC index of the class $U:=\{(K^{2}{\left|S|\right)}^{-1}{\left(\pi^{T}X_{S}\right)}^{2},∥\pi ∥=1\}$ is the VC index of the class of sets $\{\left(X_{S},t\right):(K^{2}{\left|S|\right)}^{-1}{\left(\pi^{T}X_{S}\right)}^{2}\leq t,∥\pi ∥=1,t\in R\}$. Since $\{\left(X_{S},t\right):(K^{2}{\left|S|\right)}^{-1}{\left(\pi^{T}X_{S}\right)}^{2}\leq t\}=\left\{\left(X_{S},t\right):0<{\left(K\sqrt{\left|S\right|}\right)}^{-1}\pi^{T}X_{S}\leq \sqrt{t}\right\}\cup \left\{\left(X_{S},t\right):-\sqrt{t}<{\left(K\sqrt{\left|S\right|}\right)}^{-1}\pi^{T}X_{S}\leq 0\right\}$, each set of $\{\left(X_{S},t\right):(K^{2}{\left|S|\right)}^{-1}{\left(\pi^{T}X_{S}\right)}^{2}\leq t,∥\pi ∥=1,t\in R\}$ is created by taking finite unions, intersections and complements of the basic sets $\left\{\left(X_{S},t\right):{\left(K\sqrt{\left|S\right|}\right)}^{-1}\pi^{T}X_{S}<t\right\}$. Therefore, the VC index of $\{\left(X_{S},t\right):(K^{2}{\left|S|\right)}^{-1}{\left(\pi^{T}X_{S}\right)}^{2}\leq t,∥\pi ∥=1,t\in R\}$ is of the same order as the VC index of $\left\{\left(X_{S},t\right):{\left(K\sqrt{\left|S\right|}\right)}^{-1}\pi^{T}X_{S}<t\right\}$, by Lemma 2.6.17 in [50]. Then by Theorem 2.6.7 in [50], for any probability measure *Q*, there exists some universal constant $C_{3}$ such that $N\left(\epsilon ,U,L_{2}\left(Q\right)\right)\leq {\left(C_{3}/\epsilon \right)}^{2(|S|+1)}$. Likewise, $N\left(d\epsilon ,V,L_{2}\left(Q\right)\right)\leq {\left(C_{3}/\epsilon \right)}^{2(|S|+1)}$. Given a $\beta_{S,0}\in B_{S}^{0}\left(d\right)$, for any $\beta_{S}$ in the ball $\left\{\beta_{S}:\sup_{x}|\beta_{S}^{T}x-\beta_{S,0}^{T}x|<d\epsilon \right\}$, we have $\sup_{x}\left|\sigma \left(\beta_{S}^{T}x\right)-\sigma \left(\beta_{S,0}^{T}x\right)\right|<Kd\epsilon$ by Condition (4). Let $V^{\prime }:=\left\{\sigma_{\max}^{-1}\sigma \left(\beta_{S}^{T}X_{S}\right),\beta_{S}\in B_{S}^{0}\left(d\right)\right\}$. By the definition of covering number, $N\left(Kd\epsilon ,V^{\prime },L_{2}\left(Q\right)\right)\leq {\left(C_{3}/\epsilon \right)}^{2(|S|+1)}$ Given a $\sigma (\beta_{S,0}^{T}x)$ and $\pi_{0}^{T}x$, for any $\sigma (\beta_{S}^{T}x)$ in the ball $\left\{\sigma \left(\beta_{S}^{T}x\right):\sup_{x}|\sigma \left(\beta_{S}^{T}x\right)-\sigma \left(\beta_{S,0}^{T}x\right)|\leq Kd\epsilon \right\}$ and π in the ball $\left\{\pi :\sup_{x}|{\left(\pi^{T}x\right)}^{2}-{\left(\pi_{0}^{T}x\right)}^{2}|<\epsilon \right\}$, $(\sigma_{\max}K^{2}{\left|S|\right)}^{-1}\sup_{x}|\sigma \left(\beta_{S}^{T}x\right){\left(\pi^{T}x\right)}^{2}-\sigma \left(\beta_{S,0}^{T}x\right){\left(\pi_{0}^{T}x\right)}^{2}|\leq (\sigma_{\max}^{-1}Kd+(K^{2}{\left|S|\right)}^{-1})\epsilon$. Thus, $N((\sigma_{\max}^{-1}Kd+(K^{2}{\left|S|\right)}^{-1})\epsilon ,H_{S},L_{2}\left(Q\right))\leq {\left(C_{3}/\epsilon \right)}^{4(|S|+1)}$, and consequently $N\left(\epsilon ,H_{S},L_{2}\left(Q\right)\right)\leq {\left(C_{4}/\epsilon \right)}^{4(|S|+1)}$ for some constant $C_{4}$. By Theorem 1.1 in [51] and |S|≤q, we can find some constant $C_{5}$ such that

$$\begin{array}{cc} \; & P\left(\underset{∥\pi ∥=1,\beta_{S}\in B_{S}^{0}\left(d\right)}{\sup}\left|G_{n}\left[h(\pi ,\beta_{S})\right]\right|\geq C_{5}\sqrt{q\log p}\right)\\ \leq & \frac{C_{4}^{\prime }}{C_{5}\sqrt{q\log p}}{\left(\frac{C_{4}^{\prime }C_{5}^{2}q\log p}{4(|S|+1)}\right)}^{4(|S|+1)}\exp\left(-2C_{5}^{2}q\log p\right)\\ \leq & \exp\left(4\left(|S|+1\right)\log\left(C_{4}^{\prime }C_{5}^{2}q\log p\right)-2C_{5}^{2}q\log p\right)\leq \exp\left(-5q\log p\right), \end{array}$$

where $C_{4}^{\prime }$ is some constant that depends on $C_{4}$ only. Thus,

(A5) $$\begin{array}{ccc} & & \;P\left(\underset{\left|S\right|\leq q,∥\pi ∥=1,\beta_{S}\in B_{S}^{0}\left(d\right)}{\sup}|E_{n}\left\{\sigma \left(X_{S}^{T}\beta_{S}\right){\left(\pi^{T}X_{S}\right)}^{2}\right\}-E\left[\sigma \left(X_{S}^{T}\beta_{S}\right){\left(\pi^{T}X_{S}\right)}^{2}\right]|\geq C_{5}K^{2}\sqrt{q^{3}\log p/n}\right)\\ \leq & & \;\sum_{s=|M|}^{q}{\left(\frac{ep}{s}\right)}^{s}\exp\left(-5q\log p\right)\leq \exp\left(-3q\log p\right). \end{array}$$

By Condition (5), $\sigma_{\min}\kappa_{\min}\leq \Lambda \left(E\left[\sigma \left(X_{S}^{T}\beta_{S}\right)X_{S}^{⊗2}\right]\right)\leq \sigma_{\max}\kappa_{\max}$, for all $\beta_{S}\in B_{S}^{0}\left(d\right)$ and S:M⊆S,|S|<q. Then, by (A5),

$$\begin{array}{c} \sigma_{\min}\kappa_{\min}/2\leq \Lambda \left(E_{n}\left\{\sigma \left(X_{S}^{T}\beta_{*S}\right)X_{S}^{⊗2}\right\}\right)\leq 2\sigma_{\max}\kappa_{\max} \end{array}$$

uniformly over all *S* satisfying M⊆S and |S|≤q, with probability at least 1−exp(−3qlogp). Hence, the condition (A4) in [17] is satisfied with probability at least 1−exp(−3qlogp). Additionally, for any $\beta_{S}\in B_{S}^{0}\left(d\right)$,

$$\begin{array}{cc} \; & \left|E_{n}\left\{\sigma \left(X_{S}^{T}\beta_{S}\right){\left(\pi^{T}X_{S}\right)}^{2}\right\}-E_{n}\left\{\sigma \left(X_{S}^{T}\beta_{*S}\right){\left(\pi^{T}X_{S}\right)}^{2}\right\}\right|\\ \leq & \left|n^{-1/2}G_{n}\left\{\sigma \left(X_{S}^{T}\beta_{S}\right){\left(\pi^{T}X_{S}\right)}^{2}\right\}|+|n^{-1/2}G_{n}\left\{\sigma \left(X_{S}^{T}\beta_{*S}\right){\left(\pi^{T}X_{S}\right)}^{2}\right\}\right|\\ \; & \;+|E\left[\sigma \left(X_{S}^{T}\beta_{S}\right){\left(\pi^{T}X_{S}\right)}^{2}\right]-E\left[\sigma \left(X_{S}^{T}\beta_{*S}\right){\left(\pi^{T}X_{S}\right)}^{2}\right]|\\ \leq & 2C_{5}K^{2}\sqrt{q^{3}\log p/n}+\mu_{\max}∥\beta_{S}-\beta_{*S}∥\sqrt{\left|S\right|}K\lambda_{\max}. \end{array}$$

Hence, the condition (A5) in [17] is satisfied uniformly over all *S* such that M⊆S and |S|≤q, with probability at least 1−exp(−3qlogp). Then (A4) can be shown by following the proof of Equation (3.2) in [17]. Thus, our forward stage stops at the *k*th step with probability at least 1−exp(−3qlogp). □

**Proof** **of** **Theorem 4.** Suppose that a covariate $X_{r}$ is removed from *S*. For any r∈M, since M⊈S\{r} and *r* is the only element that is in ${\left(S\backslash \left\{r\right\}\right)}^{c}\cap M$, by Lemma A1 and (A2)

$$\begin{array}{ccc} & & \;\ell_{S}\left({\hat{\beta }}_{S}\right)-\ell_{S\backslash \{r\}}\left({\hat{\beta }}_{S\backslash \{r\}}\right)\geq \ell_{S}\left(\beta_{S}^{*}\right)-\ell_{S\backslash \{r\}}\left({\hat{\beta }}_{S\backslash \{r\}}\right)\\ & & =\ell_{S\backslash \{r\}\cup \{r\}}\left(\beta_{S\backslash \{r\}+r}^{*}\right)-\ell_{S\backslash \{r\}}\left({\hat{\beta }}_{S\backslash \{r\}}\right)\geq C_{1}n^{-2\alpha }, \end{array}$$

with probability at least 1−6exp(−6qlogp). From the proof of Theorem 1, we have for any $\eta_{2}>0$, $BIC\left(S\right)-BIC\left(S\backslash \left\{r\right\}\right)\leq -2C_{1}n^{-2\alpha }+\eta_{2}n^{-1}\log n<0,$ uniformly over r∈M and *S* satisfying M⊂S and |S|≤q, with probability at least 1−6exp(−6qlogp). □

**Proof** **of** **Theorem 5.** By Theorems 1–3, we have that the event $\Omega_{1}:=\left\{\right|\hat{M}|\leq q$ and $M\subseteq \hat{M}\}$ holds with probability at least 1−25exp(−2qlogp). Thus, in the rest of the proof, we restrict our attention on $\Omega_{1}$. As shown in the proof of Theorem 3, we obtain that $\beta_{\hat{M}}^{*}=\beta_{*\hat{M}}$. Then by Lemma A6, we have $∥{\hat{\beta }}_{\hat{M}}-\beta_{\hat{M}}^{*}∥\leq A_{2}K^{-1}\sqrt{q^{2}\log p/n}$ with probability at least 1−6exp(−6qlogp). Withdrawing the attention on $\Omega_{1}$, we obtain that

$$∥\hat{\beta }-\beta_{*}∥=∥{\hat{\beta }}_{\hat{M}}-\beta_{*\hat{M}}∥=∥{\hat{\beta }}_{\hat{M}}-\beta_{\hat{M}}^{*}∥\leq A_{2}K^{-1}\sqrt{q^{2}\log p/n},$$

with probability at least 1−31exp(−2qlogp). □

### Appendix Additional Lemmas and Proofs

**Lemma** **A1.**
*Given a model S such that |S|<q,M⊈S, under Condition (6),*

*(i): $\exists r\in S^{c}\cap M$, such that $\beta_{S+r}^{*}\neq {\left(\beta_{S}^{*T},0\right)}^{T}$.*

*(ii): Suppose Conditions (1), (2) and (6’) hold. $\exists r\in S^{c}\cap M$, such that $∥\beta_{S+r}^{*T}-\left(\beta_{S}^{*T},0\right)∥\geq C\sigma_{\max}^{-1}\kappa_{\max}^{-1}n^{-\alpha }$.*

**Proof.** As $\beta_{S+j}^{*}$ is the maximizer of $E\left[\ell_{S\cup \{j\}}\left(\beta_{S+j}\right)\right]$, by the concavity of $E\left[\ell_{S\cup \{j\}}\left(\beta_{S+j}\right)\right]$, $\beta_{S+j}^{*}$ is the solution to the equation $E\left[\left(Y-\mu \left(\beta_{S}^{*T}X_{S}+\beta_{j}X_{j}\right)\right)X_{S\cup \{j\}}\right]=0$, (i): Suppose that $\beta_{S+j}^{*}={\left(\beta_{S}^{*T},0\right)}^{T},\forall j\in S^{c}\cap M$. Then,

$$\begin{array}{cc} \; & 0=E\left[\left(Y-\mu \left(\beta_{S}^{*T}X_{S}\right)\right)X_{j}\right]=E\left[\left(\mu \left(\beta_{*}^{T}X\right)-\mu \left(\beta_{S}^{*T}X_{S}\right)\right)X_{j}\right]\\ \; & \Rightarrow \max_{j\in S^{c}\cap M}|E\left[\left(\mu \left(\beta_{*}^{T}X\right)-\mu \left(\beta_{S}^{*T}X_{S}\right)\right)X_{j}\right]|=0, \end{array}$$

which violates the Condition (6). Therefore, we can find a $r\in S^{c}\cap M$, such that $\beta_{S+r}^{*}\neq {\left(\beta_{S}^{*T},0\right)}^{T}$. (ii): Let $r\in S^{c}\cap M$ satisfy that $|E\left[\left(\mu \left(\beta_{*}^{T}X\right)-\mu \left(\beta_{S}^{*T}X_{S}\right)\right)X_{r}\right]|>Cn^{-\alpha }$. Without loss of generality, we assume that $X_{r}$ is the last element of $X_{S\cup \{r\}}$. By the mean value theorem,

(A6) $$\begin{array}{ccc} & & \;E\left[\left(\mu \left(\beta_{*}^{T}X\right)-\mu \left(\beta_{S}^{*T}X_{S}\right)\right)X_{r}\right]\\ & & =E\left[\left(\mu \left(\beta_{*}^{T}X\right)-\mu \left(\beta_{S}^{*T}X_{S}\right)\right)X_{r}\right]-E\left[\left(\mu \left(\beta_{*}^{T}X\right)-\mu \left(\beta_{S+r}^{*T}X_{S\cup \{r\}}\right)\right)X_{r}\right]\\ & & =E\left[\left(\mu \left(\beta_{S+r}^{*T}X_{S\cup \{r\}}\right)-\mu \left(\left(\beta_{S}^{*T},0\right)X_{S\cup \{r\}}\right)\right)X_{r}\right]\\ & & =\left(\beta_{S+r}^{*T}-\left(\beta_{S}^{*T},0\right)\right)E\left[\sigma \left({\tilde{\beta }}_{S+r}^{T}X_{S\cup \{r\}}\right)X_{S\cup \{r\}}^{⊗2}\right]e_{r}, \end{array}$$

where ${\tilde{\beta }}_{S+r}$ is some point between $\beta_{S+r}^{*}$ and ${\left(\beta_{S}^{*T},0\right)}^{T}$ and $e_{r}$ is a vector of length (|S|+1) with the *r*th element being 1. As ${\tilde{\beta }}_{S+r}$ is some point between $\beta_{S+r}^{*}$ and ${\left(\beta_{S}^{*T},0\right)}^{T}$, $|{\tilde{\beta }}_{S+r}^{T}X_{S\cup \{r\}}\left|\leq \right|\beta_{S+r}^{*T}X_{S\cup \{r\}}\left|+\right|\left(\beta_{S}^{*T},0\right)X_{S\cup \{r\}}|\leq 2K^{2}$, by Conditions (1) and (2). Thus, $|\sigma \left({\tilde{\beta }}_{S+r}^{T}X_{S\cup \{r\}}\right)|\leq \sigma_{\max}$. By (A6) and Condition (5),

$$\begin{array}{cc} \; & \;Cn^{-\alpha }\leq \left|E\left[\left(\mu \left(\beta_{*}^{T}X\right)-\mu \left(\beta_{S}^{*T}X_{S}\right)\right)X_{r}\right]\right|\\ \; & \leq ∥\beta_{S+r}^{*T}-\left(\beta_{S}^{*T},0\right)∥\sigma_{\max}\lambda_{\max}\left(E\left[X_{S\cup \{r\}}^{⊗2}\right]\right)∥e_{r}∥\leq \sigma_{\max}\kappa_{\max}∥\beta_{S+r}^{*T}-\left(\beta_{S}^{*T},0\right)∥. \end{array}$$

Therefore, $∥\beta_{S+r}^{*T}-\left(\beta_{S}^{*T},0\right)∥\geq C\sigma_{\max}^{-1}\kappa_{\max}^{-1}n^{-\alpha }$. □

**Lemma** **A2.**
*Let $\xi_{i},i=1,\ldots ,n$ be n i.i.d random variables such that $|\xi_{i}|\leq B$ for a constant B>0. Under Conditions (1)–(3), we have $E\left[|Y_{i}\xi_{i}-E\left[Y_{i}\xi_{i}\right]{|}^{m}\right]\leq m!{\left(2B\left(\sqrt{2}M+\mu_{\max}\right)\right)}^{m}$, for every m≥1.*

**Proof.** By Conditions (1) and (2), $|\beta_{*}^{T}X_{i}|\leq KL$, ∀i≥1 and consequently $\left|\mu (\beta_{*}^{T}X_{i})\right|\leq \mu_{\max}$. Then by Condition (3),

E[|Yi|m]=E[|ϵi+μ(β*TXi)|m]≤∑t=0mmtE|ϵi|tμmaxm−t≤∑t=0mt!mtMtμmaxm−t≤m!(M+μmax)m,

for every m≥1. By the same arguments, it can be shown that, for every m≥1, $E\left[|Y_{i}\xi_{i}-E\left[Y_{i}\xi_{i}\right]{|}^{m}\right]\leq E\left[{\left(|Y_{i}\xi_{i}|+|E\left[Y_{i}\xi_{i}\right]|\right)}^{m}\right]\leq m!{\left(2B\left(M+\mu_{\max}\right)\right)}^{m}.$ □

**Lemma** **A3.**
*Under Conditions (1)–(3), when n is sufficiently large such that $28\sqrt{q\log p/n}<1$, we have $\sup_{\beta \in B}\left|E_{n}\left\{L(\beta^{T}X,Y)\right\}\right|\leq 2\left(M+\mu_{\max}\right)K^{3}+b_{\max}$, with probability 1−2exp(−10qlogp).*

**Proof.** By Conditions (2), $\sup_{\beta \in B}\left|\beta^{T}X\right|\leq K^{3}$. Thus,

$$\begin{array}{cc} \; & \;\underset{\beta \in B}{\sup}\left|E_{n}\left\{L(\beta^{T}X,Y)\right\}\right|\leq \underset{\beta \in B}{\sup}\left|E_{n}\left\{\left|Y\beta^{T}X\right|\right\}\right|+b_{\max}\\ \; & \leq \left(|E_{n}\left\{\left|Y\right|-E\left[|Y|\right]\right\}|+E\left[|Y|\right]\right)K^{3}+b_{\max}\\ \; & \leq \left(|E_{n}\left\{\left|Y\right|-E\left[|Y|\right]\right\}|\right)K^{3}+\left(M+\mu_{\max}\right)K^{3}+b_{\max}, \end{array}$$

where the last inequality follows from that $E\left[|Y|\right]\leq M+\mu_{\max}$ as shown in the proof of Lemma A2. Let $\xi_{i}=1\left\{Y_{i}>0\right\}-1\left\{Y_{i}<0\right\}$. Thus $|\xi_{i}|\leq 1$. By Lemma A2, we have $E\left[\left|\right|Y_{i}|-E\left[|Y_{i}|\right]{|}^{m}\right]\leq m!{\left(2\left(M+\mu_{\max}\right)\right)}^{m}$. Applying Bernstein’s inequality (e.g., Lemma 2.2.11 in [50]) yields that

(A7) $$\begin{array}{ccc} & & \;P\left(\left|E_{n}\left\{\left|Y\right|-E\left[|Y|\right]\right\}\right|>10\left(M+\mu_{\max}\right)\sqrt{q\log p/n}\right)\\ & & \leq 2\exp\left(-\frac{1}{2}\frac{196q\log p}{4+20\sqrt{q\log p/n}}\right)\leq 2\exp\left(-10q\log p\right), \end{array}$$

when *n* is sufficiently large such that $20\sqrt{q\log p/n}<1$. Since $10\left(M+\mu_{\max}\right)\sqrt{q\log p/n}=o\left(1\right)$, then

$$\begin{array}{c} P\left(\underset{\beta \in B}{\sup}\left|E_{n}\left\{L(\beta^{T}X,Y)\right\}\right|\geq 2\left(M+\mu_{\max}\right)K^{3}+b_{\max}\right)\leq 2\exp\left(-10q\log p\right). \end{array}$$

□

**Lemma** **A4.**
*Given an index set S and $r\in S^{c}$, let $B_{S}^{0}\left(d\right)=\{\beta_{S}:∥\beta_{S}-\beta_{S}^{*}∥\leq d/\left(K\sqrt{\left|S\right|}\right)\}$ and $A_{1}:=\left(M+2\mu_{\max}\right)$. Under Conditions (1)–(3), when n is sufficiently large such that $10\sqrt{q\log p/n}<1$, we have*

1. *$|G_{n}\left[L\left(\beta_{S}^{T}X_{S},Y\right)-L\left(\beta_{S}^{*T}X_{S},Y\right)\right]|\leq 20A_{1}d\sqrt{q\log p}$, uniformly over $\beta_{S}\in B_{S}^{0}\left(d\right)$ and |S|≤q, with probability at least 1−4exp(−6qlogp).*
2. *$|G_{n}\left[L(\beta_{S}^{*T}X_{S},Y)\right]|\leq 10\left(A_{1}K^{2}+b_{\max}\right)\sqrt{q\log p}$, uniformly over |S|≤q, with probability at least 1−2exp(−8qlogp).*

**Proof.** : (1): Let $R_{\left|S\right|}\left(d\right)$ be a |S|-dimensional ball with center at 0 and radius $d/(K\sqrt{\left|S\right|})$. Then $B_{S}^{0}\left(d\right)=R_{\left|S\right|}\left(d\right)+\beta_{S}^{*}$. Let $C_{\left|S\right|}:=\left\{C\left(\xi_{k}\right)\right\}$ be a collection of cubes that cover the ball $R_{\left|S\right|}\left(d\right)$, where $C(\xi_{k})$ is a cube containing $\xi_{k}$ with sides of length $d/(K\sqrt{\left|S\right|}n^{2})$ and $\xi_{k}$ is some point in $R_{\left|S\right|}\left(d\right)$. As the volume of $C(\xi_{k})$ is ${\left(d/(K\sqrt{\left|S\right|}n^{2})\right)}^{\left|S\right|}$ and the volume of $R_{\left|S\right|}\left(d\right)$ is less than ${\left(2d/\left(K\sqrt{\left|S\right|}\right)\right)}^{\left|S\right|}$, we can select $\xi_{k}$s so that no more than ${\left(4n^{2}\right)}^{\left|S\right|}$ cubes are needed to cover $R_{\left|S\right|}\left(d\right)$. We thus assume $|C_{\left|S\right|}|\leq {\left(4n^{2}\right)}^{\left|S\right|}$. For any $\xi \in C(\xi_{k})$, $∥\xi -\xi_{k}∥\leq d/\left(Kn^{2}\right)$. In addition, let $T_{1S}\left(\xi \right):=E_{n}\left[Y\xi^{T}X_{S}\right]$, $T_{2S}\left(\xi \right):=E_{n}\left[b\left({\left(\beta_{S}^{*}+\xi \right)}^{T}X_{S}\right)-b\left(\beta_{S}^{*T}X_{S}\right)\right]$, and $T_{S}\left(\xi \right):=T_{1S}\left(\xi \right)-T_{2S}\left(\xi \right)$. Given any $\xi \in R_{\left|S\right|}\left(d\right)$, there exists $C\left(\xi_{k}\right)\in C_{\left|S\right|}$ such that $\xi \in C(\xi_{k})$. Then

$$\begin{array}{cc} \left|T_{S}\left(\xi \right)-E\left[T_{S}\left(\xi \right)\right]\right| & \leq \left|T_{S}\left(\xi \right)-T_{S}\left(\xi_{k}\right)\right|\left|T_{S}\left(\xi_{k}\right)-E\left[T_{S}\left(\xi_{k}\right)\right]\right|+\left|E\left[T_{S}\left(\xi \right)\right]-E\left[T_{S}\left(\xi_{k}\right)\right]\right|\\ \; & =:I+II+III. \end{array}$$

We deal with III first. By the mean value theorem, there exists a $\tilde{\xi }$ between ξ and $\xi_{k}$ such that

(A8) $$\begin{array}{ccc} & & \;\left|E\left[T_{S}\left(\xi_{k}\right)\right]-E\left[T_{S}\left(\xi \right)\right]\right|=\left|E\left[Y{\left(\xi_{k}-\xi \right)}^{T}X_{S}\right]+E\left[\mu \left({\left(\beta_{S}^{*}+\tilde{\xi }\right)}^{T}X_{S}\right){\left(\xi_{k}-\xi \right)}^{T}X_{S}\right]\right|\\ & & \leq E[|Y|]∥\xi_{k}-\xi ∥∥X_{S}∥+\mu_{\max}∥\xi_{k}-\xi ∥∥X_{S}∥\leq \left(M+2\mu_{\max}\right)d\sqrt{\left|S\right|}n^{-2}=A_{1}d\sqrt{\left|S\right|}n^{-2}, \end{array}$$

where the last inequality follows from Lemma A2 and $A_{1}=M+2\mu_{\max}$. Next, we evaluate II. By Condition (2), $|X_{iS}^{T}\xi |\leq ∥X_{iS}∥∥\xi ∥\leq d/\left(K\sqrt{\left|S\right|}\right)\sqrt{\left|S\right|}K=d$, for all $\xi \in R_{\left|S\right|}\left(d\right)$. Then by Lemma A2,

$$\begin{array}{c} E\left[{\left|Y\xi_{k}^{T}X_{S}-E\left[Y\xi_{k}^{T}X_{S}\right]\right|}^{m}\right]\leq m!{\left(2\left(M+\mu_{\max}\right)d\right)}^{m}. \end{array}$$

By Bernstein’s inequality, when *n* is sufficiently large such that $10\sqrt{q\log p/n}\leq 1$.

(A9) $$\begin{array}{ccc} & & P\left(\left|T_{1S}\left(\xi_{k}\right)-E\left[T_{1S}\left(\xi_{k}\right)\right]\right|>10\left(M+\mu_{\max}\right)d\sqrt{qn^{-1}\log p}\right)\\ & & \leq 2\exp\left(-\frac{1}{2}\frac{100q\log p}{4+20\sqrt{q\log p/n}}\right)\leq 2\exp\left(-10q\log p\right). \end{array}$$

Since $|b\left({\left(\beta_{S}^{*}+\xi_{k}\right)}^{T}X_{S}\right)-b\left(\beta_{S}^{*T}X_{S}\right)|\leq \mu_{\max}d$, by the same arguments used for (A9), we have

(A10) $$\begin{array}{c} P\left(\left|T_{2S}\left(\xi_{k}\right)-E\left[T_{2S}\left(\xi_{k}\right)\right]\right|>10\mu_{\max}d\sqrt{qn^{-1}\log p}\right)\leq 2\exp\left(-10q\log p\right). \end{array}$$

Combining (A9) and (A10) yields that uniformly over $\xi_{k}$

(A11) $$\left|T_{S}\left(\xi_{k}\right)-E\left[T_{S}\left(\xi_{k}\right)\right]\right|\leq 10A_{1}d\sqrt{qn^{-1}\log p},$$

with probability at least $1-2{\left(4n^{2}\right)}^{\left|S\right|}\exp\left(-10q\log p\right)$. We now assess *I*. Following the same arguments as in Lemma A3,

(A12) $$\begin{array}{c} P\left(\underset{\xi \in C(\xi_{k})}{\sup}\left|T_{S}\left(\xi \right)-T_{S}\left(\xi_{k}\right)\right|>\left(2M+3\mu_{\max}\right)d\sqrt{\left|S\right|}n^{-2}\right)\leq 2\exp\left(-8q\log p\right). \end{array}$$

Since $\sqrt{\left|S\right|}n^{-2}=o\left(\sqrt{qn^{-1}\log p}\right)$, combining (A8), (A11) and (A12) together yields that

$$\begin{array}{cc} \; & \;P\left(\underset{\xi \in R_{\left|S\right|}\left(d\right)}{\sup}\left|T_{S}\left(\xi \right)-E\left[T_{S}\left(\xi \right)\right]\right|\geq 20A_{1}d\sqrt{qn^{-1}\log p}\right)\\ \; & \leq 2{\left(4n^{2}\right)}^{\left|S\right|}\exp\left(-10q\log p\right)+2\exp\left(-8q\log p\right)\leq 4\exp\left(-8q\log p\right). \end{array}$$

By the combinatoric inequality ps≤(ep/s)s, we obtain that

$$\begin{array}{cc} \; & \;P\left(\underset{\left|S\right|\leq q,\beta_{S}\in B_{S}^{0}\left(d_{1}\right)}{\sup}\left|G_{n}\left[L\left(\beta_{S}^{T}X_{S},Y\right)-L\left(\beta_{S}^{*T}X_{S},Y\right)\right]\right|\geq 20A_{1}d\sqrt{q\log p}\right)\\ \; & \leq \sum_{s=1}^{q}{\left(ep/s\right)}^{s}4\exp\left(-8q\log p\right)\leq 4\exp\left(-6q\log p\right). \end{array}$$

(2): We evaluate the *m*th moment of $L(\beta_{S}^{*}X_{S},Y)$.

EYβS*XS−b(βS*XS)m≤E∑t=0mmt|Y|tK2tbmaxm−t≤∑t=0mmtt!(M+μmax)K2tbmaxm−t≤m!((M+μmax)K2+bmax)m.

Then, by Bernstein’s inequality,

$$\begin{array}{c} P\left(|G_{n}\left[L(\beta_{S}^{*T}X_{S},Y)\right]|>10\left(A_{1}K^{2}+b_{\max}\right)\sqrt{q\log p}\right)\leq 2\exp\left(-10q\log p\right). \end{array}$$

By the same arguments used in (i), we obtain that

$$\begin{array}{cc} \; & \;P\left(\underset{|S|\leq q}{\sup}\left|G_{n}\left[L\left(\beta_{S}^{*T}X_{S},Y\right)\right]\right|\geq 10\left(A_{1}K^{2}+b_{\max}\right)\sqrt{q\log p}\right)\\ \; & \leq \sum_{s=1}^{q}{\left(ep/s\right)}^{s}2\exp\left(-10q\log p\right)\leq 2\exp\left(-8q\log p\right). \end{array}$$

□

**Lemma** **A5.**
*Given a model S and $r\in S^{c}$, under Conditions (1), (2) and (5), for any $∥\beta_{S}-\beta_{S}^{*}∥\leq K/\sqrt{\left|S\right|}$, $\sigma_{\min}\kappa_{\min}∥\beta_{S}-\beta_{S}^{*}{∥}^{2}/2\leq E\left[\ell_{S}\left(\beta_{S}^{*}\right)\right]-E\left[\ell_{S}\left(\beta_{S}\right)\right]\leq \sigma_{\max}\kappa_{\max}{∥\beta_{S}-\beta_{S}^{*}∥}^{2}/2$.*

**Proof.** Due to the concavity of the log-likelihood in GLMs with the canonical link, $E\left[YX_{S}-\mu \left(\beta_{S}^{*T}X_{S}\right)X_{S}\right]=0$. Then for any $∥\beta_{S}-\beta_{S}^{*}∥\leq K/\sqrt{\left|S\right|}$, $|\beta^{T}X_{S}\left|\leq \right|\beta^{*T}X_{S}\left|+\right|{\left(\beta -\beta^{*}\right)}^{T}X_{S}|\leq K^{2}+K/\sqrt{\left|S\right|}\times K\sqrt{\left|S\right|}=2KL$. Thus, by Taylor’s expansion,

$$\begin{array}{c} E\left[\ell_{S}\left(\beta_{S}\right)\right]-E\left[\ell_{S}\left(\beta_{S}^{*}\right)\right]=-\frac{1}{2}{\left(\beta_{S}-\beta_{S}^{*}\right)}^{T}E\left[\sigma \left({\tilde{\beta }}_{S}^{T}X_{S}\right)X_{S}^{⊗2}\right]\left(\beta_{S}-\beta_{S}^{*}\right), \end{array}$$

where ${\tilde{\beta }}_{S}$ is between $\beta_{S}$ and $\beta_{S}^{*}$. By Condition (5), $\sigma_{\min}\kappa_{\min}∥\beta_{S}-\beta_{S}^{*}{∥}^{2}/2\leq E\left[\ell_{S}\left(\beta_{S}^{*}\right)\right]-E\left[\ell_{S}\left(\beta_{S}\right)\right]\leq \sigma_{\max}\kappa_{\max}{∥\beta_{S}-\beta_{S}^{*}∥}^{2}/2.$ □

**Lemma** **A6.**
*Under Conditions (1)–(6), there exist some constants $A_{2}$ and $A_{3}$ that do not depend on n, such that $∥{\hat{\beta }}_{S}-\beta_{S}^{*}∥\leq A_{2}K^{-1}\sqrt{q^{2}\log p/n}$ and $|\ell_{S}\left({\hat{\beta }}_{S}\right)-\ell_{S}\left(\beta_{S}^{*}\right)|\leq A_{3}q^{2}\log p/n$ hold uniformly over S:|S|≤q, with probability at least 1−6exp(−6qlogp).*

**Proof.** Define

$$\begin{array}{cc} \; & \Omega \left(d\right):=\left\{\underset{\left|S\right|\leq q,\beta_{S}\in B_{S}^{0}\left(d\right)}{\sup}|G_{n}\left[L\left(\beta_{S}^{T}X_{S},Y\right)-L\left(\beta_{S}^{*T}X_{S},Y\right)\right]|<20A_{1}d\sqrt{q\log p}\right\}. \end{array}$$

By Lemma A4, the event Ω(d) holds with probability at least 1−4exp(−6qlogp). Thus, in the proof of Lemma A6, we shall assume Ω(d) hold with $d=A_{2}\sqrt{q^{3}\log p/n}$ for some $A_{2}>20{\left(\sigma_{\min}\kappa_{\min}\right)}^{-1}K^{2}A_{1}$. For any $∥\beta_{S}-\beta_{S}^{*}∥=A_{2}K^{-1}\sqrt{q^{2}\log p/n}$, since $\sqrt{q^{2}\log p/n}$$\leq \sqrt{q^{3}\log p/n}/\sqrt{\left|S\right|}$, $\beta_{S}\in B_{S}^{0}\left(d\right)$. By Lemma A5,

$$\begin{array}{cc} \; & \;\ell_{S}\left(\beta_{S}^{*}\right)-\ell_{S}\left(\beta_{S}\right)\\ \; & =\left(\ell_{S}\left(\beta_{S}^{*}\right)-E\left[\ell_{S}\left(\beta_{S}^{*}\right)\right]-\left(\ell_{S}\left(\beta_{S}\right)-E\left[\ell_{S}\left(\beta_{S}\right)\right]\right)\right)+\left(E\left[\ell_{S}\left(\beta_{S}^{*}\right)\right]-E\left[\ell_{S}\left(\beta_{S}\right)\right]\right)\\ \; & \geq \sigma_{\min}\kappa_{\min}{∥\beta_{S}-\beta_{S}^{*}∥}^{2}/2-20A_{1}d\sqrt{q\log p/n}\\ \; & =\sigma_{\min}\kappa_{\min}A_{2}^{2}q^{2}\log p/\left(K^{2}n\right)-20A_{1}A_{2}q^{2}\log p/n>0. \end{array}$$

Thus,

$$\underset{|S|\leq q,∥\beta_{S}-\beta_{S}^{*}∥=A_{2}K^{-1}\sqrt{q^{2}\log p/n}}{\inf}\ell_{S}\left(\beta_{S}^{*}\right)-\ell_{S}\left(\beta_{S}\right)>0.$$

Then by the concavity of $\ell_{S}\left(\;\cdot \;\right)$, we obtain that $\max_{|S|\leq q}∥{\hat{\beta }}_{S}-\beta_{S}^{*}∥\leq A_{2}K^{-1}\sqrt{q^{2}n^{-1}\log p}$. On the other hand, for any $∥\beta_{S}-\beta_{S}^{*}∥\leq A_{2}K^{-1}\sqrt{q^{2}\log p/n}$,

$$\begin{array}{cc} \; & \;\left|\ell_{S}\left(\beta_{S}^{*}\right)-\ell_{S}\left(\beta_{S}\right)\right|\\ \; & \leq |\ell_{S}\left(\beta_{S}^{*}\right)-E\left[\ell_{S}\left(\beta_{S}^{*}\right)\right]-\left(\ell_{S}\left(\beta_{S}\right)-E\left[\ell_{S}\left(\beta_{S}\right)\right]\right)|+\left|E\left[\ell_{S}\left(\beta_{S}^{*}\right)\right]-E\left[\ell_{S}\left(\beta_{S}\right)\right]\right|\\ \; & \leq \sigma_{\max}\kappa_{\max}{∥\beta_{S}-\beta_{S}^{*}∥}^{2}/2+20A_{1}d\sqrt{q\log p/n}\leq A_{3}q^{2}n^{-1}\log p, \end{array}$$

where $A_{3}:=4\sigma_{\max}\lambda_{\max}A_{2}^{2}K^{-2}+20A_{1}A_{2}.$ □

## Appendix B. Additional Results in the Applications

**Table A1:** Comparison of genes selected by each competing method from the mammalian eye data set.

	STEPWISE	FR	LASSO	SIS+LASSO	SC	dgLARS
STEPWISE	3	3	2	2	2	0
FR		4	2	2	2	0
LASSO			16	5	2	0
SIS+LASSO				9	2	0
SC					4	0
dgLARS						7

Note: Diagonal and off-diagonal elements of the table represent the model sizes for each method and the number of overlapping genes selected by the two methods corresponding to the row and column, respectively.

**Table A2:** Selected miRNAs for ESCC training dataset.

Methods	Selected miRNAs
STEPWISE	*miR-4783-3p*; *miR-32*0b; *miR-1225-3p*
FR	*miR-4783-3p*; *miR-320b*; *miR-1225-3p*; *6789-5p*
SC	*miR-4783-3p*; *miR-320b*; *miR-1225-3p*; *6789-5p*
LASSO	*miR-6789-5p*; *miR-6781-5p*; *miR-1225-3p*; *miR-1238-5p*; *miR-320b*;
	*miR-6794-5p*; *miR-6877-5p*; *miR-6785-5p*; *miR-718*; *miR-195-5p*
SIS+LASSO	*miR-6785-5p*; *miR-1238-5p*; *miR-1225-3p*; *miR-6789-5p*; *miR-320b*;
	*miR-6875-5p*; *miR-6127*; *miR-1268b*; *miR-6781-5p*; *miR-125a-3p*
dgLARS	*miR-891b*; *miR-6127*; *miR-151a-5p*; *miR-195-5p*; ; *miR-3688-5p*
	*miR-125b-1-3p*; *miR-1273c*; *miR-6501-5p*; *miR-4666a-5p*; *miR-514a-3p*

Note: LASSO, SIS+LASSO, dgLARS selected 20, 17 and 33 miRNAs, respectively, and we only reported top 10 miRNAs.

## References

[1] Prosperi M., Min J.S., Bian J., Modave F. (2018). Big data hurdles in precision medicine and precision public health. BMC Med. Inform. Decis. Mak..

[2] Tibshirani R. (1996). Regression shrinkage and selection via the lasso. J. R. Stat. Soc. Ser. B-Stat. Methodol..

[3] Flynn C.J., Hurvich C.M., Simonoff J.S. (2017). On the sensitivity of the lasso to the number of predictor variables. Stat. Sci..

[4] van de Geer S.A. (2019). On the asymptotic variance of the debiased Lasso. Electron. J. Stat..

[5] Fan J., Lv J. (2008). Sure independence screening for ultrahigh dimensional feature space (with discussion). J. R. Stat. Soc. Ser. B-Stat. Methodol..

[6] Barut E., Fan J., Verhasselt A. (2016). Conditional sure independence screening. J. Am. Stat. Assoc..

[7] Wang H. (2009). Forward regression for ultra-high dimensional variable screening. J. Am. Stat. Assoc..

[8] Zheng Q., Hong H.G., Li Y. (2019). Building generalized linear models with ultrahigh dimensional features: A sequentially conditional approach. Biometrics.

[9] Hong H.G., Zheng Q., Li Y. (2019). Forward regression for Cox models with high-dimensional covariates. J. Multivar. Anal..

[10] Efron B., Hastie T., Johnstone I., Tibshirani R. (2004). Least angle regression. Ann. Stat..

[11] Augugliaro L., Mineo A.M., Wit E.C. (2013). Differential geometric least angle regression: A differential geometric approach to sparse generalized linear models. J. R. Stat. Soc. Ser. B-Stat. Methodol..

[12] Pazira H., Augugliaro L., Wit E. (2018). Extended differential geometric LARS for high-dimensional GLMs with general dispersion parameter. Stat. Comput..

[13] An H., Huang D., Yao Q., Zhang C.H. (2008). Stepwise Searching for Feature Variables in High-Dimensional Linear Regression. http://eprints.lse.ac.uk/51349/.

[14] Ing C.K., Lai T.L. (2011). A stepwise regression method and consistent model selection for high-dimensional sparse linear models. Stat. Sin..

[15] Hwang J.S., Hu T.H. (2015). A stepwise regression algorithm for high-dimensional variable selection. J. Stat. Comput. Simul..

[16] McCullagh P. (1989). Generalized Linear Models.

[17] Chen J., Chen Z. (2012). Extended BIC for small-*n*-large-*P* sparse GLM. Stat. Sin..

[18] Bühlmann P., Yu B. (2006). Sparse boosting. J. Mach. Learn. Res..

[19] van de Geer S.A. (2008). High-dimensional generalized linear models and the lasso. Ann. Stat..

[20] Chen J., Chen Z. (2008). Extended Bayesian information criteria for model selection with large model spaces. Biometrika.

[21] Fan Y., Tang C.Y. (2013). Tuning parameter selection in high dimensional penalized likelihood. J. R. Stat. Soc. Ser. B-Stat. Methodol..

[22] Cheng M.Y., Honda T., Zhang J.T. (2016). Forward variable selection for sparse ultra-high dimensional varying coefficient models. J. Am. Stat. Assoc..

[23] Zhao S.D., Li Y. (2012). Principled sure independence screening for Cox models with ultra-high-dimensional covariates. J. Multivar. Anal..

[24] Kwemou M. (2016). Non-asymptotic oracle inequalities for the Lasso and group Lasso in high dimensional logistic model. ESAIM-Prob. Stat..

[25] Jiang Y., He Y., Zhang H. (2016). Variable selection with prior information for generalized linear models via the prior LASSO method. J. Am. Stat. Assoc..

[26] Zhang C.H., Huang J. (2008). The sparsity and bias of the Lasso selection in high-dimensional linear regression. Ann. Stat..

[27] Fan J., Song R. (2010). Sure independence screening in generalized linear models with NP-dimensionality. Ann. Stat..

[28] Luo S., Chen Z. (2014). Sequential Lasso cum EBIC for feature selection with ultra-high dimensional feature space. J. Am. Stat. Assoc..

[29] Luo S., Xu J., Chen Z. (2015). Extended Bayesian information criterion in the Cox model with a high-dimensional feature space. Ann. Inst. Stat. Math..

[30] Hastie T., Tibshirani R., Friedman J. (2009). The Elements of Statistical Learning: Data mining, Inference, and Prediction.

[31] Simon N., Friedman J., Hastie T., Tibshirani R. (2011). Regularization Paths for Cox’s Proportional Hazards Model via Coordinate Descent. J. Stat. Softw..

[32] Breheny P., Huang J. (2011). Coordinate descent algorithms for nonconvex penalized regression, with applications to biological feature selection. Ann. Appl. Stat..

[33] Wang X., Leng C. (2016). R Package: Screening. https://github.com/wwrechard/screening.

[34] Augugliaro L., Mineo A.M., Wit E.C. (2014). dglars: An R Package to Estimate Sparse Generalized Linear Models. J. Stat. Softw..

[35] Scheetz T.E., Kim K.Y.A., Swiderski R.E., Philp A.R., Braun T.A., Knudtson K.L., Dorrance A.M., DiBona G.F., Huang J., Casavant T.L. (2006). Regulation of gene expression in the mammalian eye and its relevance to eye disease. Proc. Natl. Acad. Sci. USA.

[36] Chiang A.P., Beck J.S., Yen H.J., Tayeh M.K., Scheetz T.E., Swiderski R.E., Nishimura D.Y., Braun T.A., Kim K.Y.A., Huang J. (2006). Homozygosity mapping with SNP arrays identifies TRIM32, an E3 ubiquitin ligase, as a Bardet–Biedl syndrome gene (BBS11). Proc. Natl. Acad. Sci. USA.

[37] He S., Peng J., Li L., Xu Y., Wu X., Yu J., Liu J., Zhang J., Zhang R., Wang W. (2019). High expression of cytokeratin CAM5.2 in esophageal squamous cell carcinoma is associated with poor prognosis. Medicine.

[38] Li B.X., Yu Q., Shi Z.L., Li P., Fu S. (2015). Circulating microRNAs in esophageal squamous cell carcinoma: Association with locoregional staging and survival. Int. J. Clin. Exp. Med..

[39] Sudo K., Kato K., Matsuzaki J., Boku N., Abe S., Saito Y., Daiko H., Takizawa S., Aoki Y., Sakamoto H. (2019). Development and validation of an esophageal squamous cell carcinoma detection model by large-scale microRNA profiling. JAMA Netw. Open.

[40] Zhang Y. (2013). Epidemiology of esophageal cancer. World J. Gastroenterol.

[41] Mathieu L.N., Kanarek N.F., Tsai H.L., Rudin C.M., Brock M.V. (2014). Age and sex differences in the incidence of esophageal adenocarcinoma: Results from the Surveillance, Epidemiology, and End Results (SEER) Registry (1973–2008). Dis. Esophagus.

[42] Zhou J., Zhang M., Huang Y., Feng L., Chen H., Hu Y., Chen H., Zhang K., Zheng L., Zheng S. (2015). MicroRNA-320b promotes colorectal cancer proliferation and invasion by competing with its homologous microRNA-320a. Cancer Lett..

[43] Lieb V., Weigelt K., Scheinost L., Fischer K., Greither T., Marcou M., Theil G., Klocker H., Holzhausen H.J., Lai X. (2018). Serum levels of miR-320 family members are associated with clinical parameters and diagnosis in prostate cancer patients. Oncotarget.

[44] Mullany L.E., Herrick J.S., Wolff R.K., Stevens J.R., Slattery M.L. (2016). Association of cigarette smoking and microRNA expression in rectal cancer: Insight into tumor phenotype. Cancer Epidemiol..

[45] Zheng H., Zhang F., Lin X., Huang C., Zhang Y., Li Y., Lin J., Chen W., Lin X. (2016). MicroRNA-1225-5p inhibits proliferation and metastasis of gastric carcinoma through repressing insulin receptor substrate-1 and activation of *β*-catenin signaling. Oncotarget.

[46] R Core Team (2018). R: A Language and Environment for Statistical Computing.

[47] Wickham H. (2016). ggplot2: Elegant Graphics for Data Analysis.

[48] Zhao P., Yu B. (2006). On model selection consistency of Lasso. J. Mach. Learn. Res..

[49] Bühlmann P., Van De Geer S. (2011). Statistics for High-dimensional Data: Methods, Theory and Applications.

[50] Vaart A.W., Wellner J.A. (1996). Weak Convergence and Empirical Processes: With Applications to Statistics.

[51] Talagrand M. (1994). Sharper bounds for Gaussian and empirical processes. Ann. Probab..

